# Global overview of the management of acute cholecystitis during the COVID-19 pandemic (CHOLECOVID study)

**DOI:** 10.1093/bjsopen/zrac052

**Published:** 2022-05-04

**Authors:** CHOLECOVID Collaborative, Harry V M Spiers, Omar Kouli, Waheed U Ahmed, Rebecca Varley, Daniel Ahari, Leah Argus, Kenneth A McLean, Sivesh K Kamarajah, Peter Coe, Ewen A Griffiths, Anthony KC Chan, Christian Macutkiewicz, Saurabh Jamdar, Michael Wilson, Catherine Fullwood, Giles Toogood, Ajith K Siriwardena, Omar Kouli, Kenneth McLean, Catherine Fullwood, Daniel Ahari, Leah Argus, Rebecca Varley, Harry V M Spiers, Omar Kouli, Waheed Ahmed, Andrew Gilchrist, Matthew Goldsworthy, Majid Rashid, P Pockney, J Varela, N Brindl, J Ramirez, C Marafante, Y Iwao, A Ghzawi, M Elhadi, H Gacaferi, C Varghese, A Adeyeye, O Alser, C Teh, M Prieto, A Hasan, H Al-Naggar, R Salgado, F Veracierto, T Lancelotti, D Solinas, R Oddi, FW Garcia, E Mazza Diez, MR Andrade Ramirez, R Bracco, D Fernandez, MA Maraschio, L Obeide, E Giordano, A Alcaraz, MA Marani, N Aguirre, F Luna, M Francesconi, F Chiham, R Ramos Cossio, FA Alvarez, DA Pantoja Pachajoa, F Mandojana, IG Merlo, MH Gonzalez, G Cervelo, R Puma, GF Vardaro, A Davis, D Jurat, C Guenoff, K Raubenheimer, K Goddard, K Brown, KJ Wegrecki, HYC Cheung, M Yang, H Cheung, J Siddiqui, JH Ahn, R Huynh, YH Lam, M Afzal, BS Ong, MYM Chua, K Ly, JE Thomson, D Watson, AC Dawson, A Drane, S Van Ruyven, EWY Lun, P Pockney, M Ferguson, JY Jeong, C De Silva, V Wills, J Gundara, E Mccourt, C Bong, R Tabone, WJ Wong, A Gray, D Koh, M Pollock, S Singhal, R Smith, NN Dudi-Venkata, H Kanhere, C Stranz, W Seow, LT Mansour, J Wormald, BPT Loveday, B Thomson, T O’Donnell, N Milenkovski, M Herath, M Trochsler, A Farfus, G Maddern, Z Bunjo, LL Kuan, G Atanasov, A Dawson, A Drane, S van Ruyven, E Lun, E Samadov, I Namazov, M Asgarov, A Ibrahimli, M Srinivasan, MF Saeed, H Aljawder, I Juma, FJ Coimbra, N Marques, WA Casteleins, A Petruzziello, G Jabur, JFP Rodriguez, PL Buso, S MacKenzie, M Hsiao, I Sljivic, A Tecson, PJ Karanicolas, R Roke, J Moon, EV Butler, F Riquelme, M Yanez, F Catan, M Uribe, F Carriel, F Oppliger, A Paredes, D Daroch, JC Aguayo, CJ Perez Rivera, LM Acosta Buitrago, A Kadamani Abiyomaa, MS Mosquera Paz, P Cabrera, J Corso, N Ozcay, A Ozant, K Arslan, H Besim, H Almezghwi, AY Azzam, S Bessa, I El-Sayes, A Badawy, M Wael, A El-Gendi, MA Azab, M Fayed, M El Kassas, M Gamal, A Tawheed, A Al Shafie, S Emile, A Elfallal, H Elfeki, M Shalaby, A Sakr, M Elbahnasawy, M Shama, W Abdel-Elsalam, S Abd-Elsalam, JE Escobar Dominguez, F Medrano, S Gaitan, OM Escalon Gonzalez, JC Alfaro Varela, M Cea, M Interiano, B Cabrera, Z Lakkis, P Georges, C Antonot, J Magnin, C Kamphues, JC Lauscher, C Schineis, FN Loch, LD Lee, K Beyer, K Bouchagier, I Galanis, D Bartziotas, E Lostoridis, P Tourountzi, EA Nagorni, A Charalabopoulos, E Baili, E Kyros, I Vagios, A Skotsimara, T Liakakos, A Alexandrou, A Papalampros, V Papadopolous, A Tooulias, I Kentarchos, C Christou, G Tsoulfas, LF Tale-Rosales, I Lopez Muralles, H Melendez, G Bran, FA Monroy Mahecha, JR Contreras, DE Porras, E Paiz, ER Soto, JR Ixcayau Hernandez, A Gupta, D Rajput, N Kumar, R Mani, R Kant, AA Sonkar, A Anand, MK Agrawal, K Gaurav, M Tripathi, S Sikora, K Bharathy, M Kumar Rangapa, DS Khuller, SK K, R Bhojwwani, S Ayyar, N Jain, A Mehraj, F Hussain, I Nazir, M Shah, NA Chowdri, A Hilmi, G Argenio, P Atelli, E Palladino, MF Armellino, N Tamini, LC Nespoli, L Degrate, M Angrisani, F Carissimi, P Bordoni, F Fleres, P Bordoni, G Clarizia, A Spolini, M Franzini, E Cucinotta, G Badessi, C Mazzeo, F Viscosi, G Pintabona, T Campagnaro, E Poletto, G Turri, A Ruzzenente, S Conci, A Guglielmi, C Feo, N Fabbri, M Fazzin, S Giaccari, CV Feo, M Massani, P Pelizzo, M Colella, R Tutino, F Cappellacci, F Medas, GL Canu, E Erdas, PG Calò, A Porcu, T Perra, AM Scanu, CF Feo, A Fancellu, P Germani, C Giunta, A Biloslavo, H Abdallah, G Aizza, A Barberis, F Belli, M Santoliquido, M Filauro, G Canonico, T Nelli, C Di Martino, L Capezzuoli, A Anastasi, L Bressan, S Cortinovis, C Nagliati, F Colombo, L Ferrario, A Bondurri, C Guerci, A Maffioli, F Catena, G Perrone, M Giuffrida, A Morini, A Annicchiarico, G Gallo, A Carpino, F Ferrari, G De Paola, G Sammarco, C Callari, L Licari, V Sorce, D Di Miceli, F Lovisetto, S Zonta, F Lovisetto, A Chessa, A Fiorini, A De Manzoni Garberini, E Angelini, C Marafante, E Moggia, A Murgese, S Mungo, SL Birolo, M Garino, NS Pipitone Federico, A Muratore, EG Lunghi, M Calabro, P Cianci, R Enrico, S Capuzzolo, L Cafagna, M Minafra, D Sasia, A Gattolin, M Migliore, R Rimonda, E Travaglio, G De Marco, C Elter, T Bargellini, S D’amico, D Zambonin, A Caponi, G Calini, A Puggioni, V Bresadola, T Zalla, S Cantafio, F Feroci, L Romoli, R Giudicissi, A Picciariello, V Papagni, R Dibra, A Picciariello, DF Altomare, E Pinotti, M Montuori, G Baronio, V Tonini, L Sartarelli, A Gori, M Cervellera, P Lapolla, P Sapienza, G Brachini, B Cirillo, M Zambon, A Mingoli, A Pascariello, L Boccia, S Benedetti, G Mantovani, M De Angelis, F Ferrara, V Testa, F Borghi, F Maione, V Pruiti Ciarello, G Giraudo, F Agresta, G Cestaro, D Prando, F Cavallo, M Zese, N Cillara, R Sechi, R Cardia, A Cannavera, G Putzu, F Frongia, A Pisanu, D Delogu, G Esposito, M Podda, A Iossa, F De Angelis, C Boru, G. Silecchia, GM Palini, G Garulli, S Veneroni, P Tammaro, P Maida, PA Leake, MG Wanliss, Y Iwao, K Sato, N Chiyonobu, H Imamura, S Yamazaki, M Watanabe, A Qasem, F Ayasra, S Al Dahabrh, A Khaled, S Alsaafin, A Al-Thunaibat, D Olaywah, S Alqudah, S Alqawasmi, A Khamees, A Guboug, M Es Salim, T Althwabteh, H Bani Khaled, N El-Hammuri, A Aljesrawi, F Alamaadany, M Eljareh, AEJ Al Gasi, S Alsaeiti, AS Alkhafeefi, T Suliaman, AHA Alanasri, ABA Haroun, A Haron, AI Kilani, M Ahmed, M Alawami, A Alawami, M Albashri, M Abusannuga, A Malek, N Jwaili, A Aldenfria, N Jwaili, F Elzwawi, A Almugaddami, ASA Egdeer, M Masoud, B Alazzabi, B Alezabi, A Shuwayyah, AAS Alkamkhe, I Aboulqasim, H Atiyah, RAA Alfagi, A Abdulmula, A Bouhuwaish, A Samer, R Salim, H Aboazamazem, B Almiqlash, M Biala, W Alganimi, R Ghamgh, N Ben Omar, A Alsoufi, M Aldreawi, N Saleim, F Sowan, H Saleem, Aqueelah Ahmed, NE Samalavicius, O Aliosin, S Dailidenas, A Dulskas, B Buckus, Z Kuliesius, R Bradunaite, I Dominguez-Rosado, GA Buerba, OE Posadas-Trujillo, A Alfaro-Goldaracena, R Cortes, MA Mercado, JL Beristain-Hernandez, VS Mora-Munoz, JM Mena-Bedolla, AR Palacios Ramirez, MM Astorga Medina, G Van Aert, S Ombashi, R Spillenaar Bilgen, D Vos, M Besselink, V Alberts, O Busch, W Bemelman, M Boermeester, F Daams, M Gordinou De Gouberville, P Van Duijvendijk, M De Graaff, J Baaij, S Gans, K Bos, B Goudsmit, B Den Dekker, A Braat, A Kuijpers, S Breukers, I Borel Rinkes, D Andel, T Hayes, D Carson, S Bhat, J van der Have, C Anderson, I Bissett, J Windsor, BM Elliott, H Scowcroft, J Mclauchlan, D Ritchie, F Jeffery, S Connor, W Xu, C Varghese, H Mashlan, V Thirayan, J Ly, MJ Mcguinness, L Ferguson, I Watt, C Harmston, A Akinmade, A Adeyeye, E Enoch, V Kayode-Nissi, I Ogundele, BA Ayoade, A Adekoya, C Nwokoro, A Opadeyi, A Adeyeye, A Yusuf, A Ojajuni, O Adepoju, Maigatari Muhammad Dauda, Musa Keffi Mubarak, Khalid lawal, Daniyan Muhammad, D Salonga, NA Sael, CM Rey, M Pestano, D Tan, NR Bangayan, DK Sy, D Ang, E Bernardo, JP Chua, M Alharthi, W Bukhari, K Bakier Mohammed, S Al Athath, M Ghunaim, H Saiedi, N Sultan, A Farsi, M Basendowah, M Alharthi, M Ghunaim, N Malibary, H Jaloun, Db Altalhi, A Organjee, M Moamena, TM Al Zaidi, M Alyami, M Alqannas, M Al-Urfan, A Elawad, A Alawadhi, Y Alalawi, A Alqarni, B Alqahtani, A Alayed, K Alsobaie, H Adi, N Malibary, M Elhaj, A Dehlawi, G Behairy, I Khaled, S Kmezic, D Radenkovic, L Aleksic, V Markovic, I Pejovic, A Antic, M Kalkan, O Vujanovic Gadjanski, S Dusan, B Marčetić, N Thiruchelvam, AKH Chiow, LS Lee, DYC Mun, EK Tan, YX Koh, WL Loh, Z Wang, CY Chan, C Kloppers, N Almgla, M Bernon, M Kahn, N Karimbocus, JI Roldan Villavicencio, V Goitia, RD Gutierrez Rios, S Garcia Ruiz, M Lopez Deogracias, V Turrado-Rodriguez, X Morales, A Hessheimer, R Termes Serra, J Beltran De Heredia, J Trujillo-Diaz, J Herreros-Rodríguez, M Montes-Manrique, B De Andres-Asenjo, J Beltrán-Heredia, T Gimenez Maurel, A Utrilla Fornals, LF Martin Anoro, S Cortese, MD Perez Diaz, M Ballón, M Morote, L Cebolla Rojas, JR Oliver Guillen, A Lopez De Fernandez, M Del Campo Lavilla, I Mora-Guzmán, A Escartin, A Pinillos, FF Vela Polanco, JH Jara Quezada, P Muriel Alvarez, J Tur-Martinez, J Camps, E Herrero, MI Garcia-Domingo, E Cugat Andorra, A Crespi Mir, O Claramonte Bellmunt, JC Vicens Arbona, IR Fernandez Burgos, M Prieto, A Sarriugarte Lasarte, H Marin, M Tellaeche De La Iglesia, O Ocerin Alganza, J Salinas Gomez, P Ramos-Martin, A Urbieta, R Nasimi Sabbagh, JT Castell Gomez, A Serrablo, S Paterna -Lopez, M Gutiérrez-Díez, MT Abadía-Forcen, M Serradilla-Martín, VM Duran Muñoz-Cruzado, F Pareja Ciuro, E Perea Del Pozo, D Aparicio Sanchez, S Dios-Barbeito, B Marenco De La Cuadra, M Retamar Gentil, J Reguera-Rosal, M Infantes Ormad, JA Lopez-Ruiz, A Landaluce-Olavarria, JC Zevallos-Quiroz, J Barrutia Leonardo, A Emaldi, E Begona, I Balciscueta Coltell, M Sebastian, S Martinez Ramos, S Martinez Alcaide, J Lorenzo Perez, LA Martinez Insfran, P Lopez-Morales, C Gimenez Frances, A Rahy-Martin, M Pelloni, D Ortiz-Lopez, O Benet-Muñoz, L Pinero-Gonzalez, F Alconchel, T Nicolas-Lopez, K Rodrigues, PA Cascales Campos, F Gomez-Bosch, P Ramirez Romero, M Ibrahim, HKS Hamid, R Idres, M Idris, O Mohammed, S Ayran, AH Sinan, O Kouli, V Ozben, E Aytac, Z Aliyeva, AU Mutlu, IA Bilgin, T Karahasanoglu, I Hamzaoglu, B Bozkirli, TK Uprak, T Kotan, M Coskun, Y Kara, E Somuncu, A Kocatas, MA Bozkurt, S Demirli Atici, T Kaya, I Sert, M Emiroglu, M Jaffar, MU Younis, T Aziz, F Ikram, M Sandal, F Al Madhloum Al Suwaidi, MO Alshaikh, A Saber, A Khammas, A Nessa, R Jardine, L Nicol, C Clark, A Mcgee, B Alkari, M Feretis, R Antakia, F Georgiades, J Moneim, R O’Neill, A Balakrishnan, R Lunevicius, A Sud, I Moutsos, D Gomez, S Shahid, T Majeed, WKG Ibrahim, K Kadum, R Melia, C Magee, DW Chicken, S Kumar, M Alshibshoubi, S van Laarhoven, F Dewi, J Williams, B Byrne, P Wilkerson, CB Tang, N Farhangmehr, A Jonas, V Charavanamuttu, K Almeida, E Efthimiou, J Boardley, A White, MA Butt, D Menzies, Z Gundkalli, D Hassanzadeh-Baboli, O Jones, P Mistry, S Saha, A Gerrard, J Evans, S Rajeev, W Ali, E Ross, A Gilliam, C Hitchins, K Emslie, K Spellar, H Sked, C Briggs, L Brown, N A Hemadasa, JR Apollos, A Belgaumkar, A Tawfik, L Brewin, B Oyewole, H Wadhawan, E Massie, D Rutherford, K Mcgivern, L Mcelroy, HD De’Ath, M Tobbal, S Nagendram, P Patel, S Handa, G Houghton, SS Sundaralingam, J Parker, R Morgan, T Gala, S Ibrahim, R Harby, M Abdelkarim, D Holroyd, D Carson, R Thomas, E Mclennan, R Boardley, NB Jamieson, H Ebied, J Gossage, A Davies, S Wheatstone, Z Jawad, L Jiao, P Rajagopal, M Sodergren, M Lami, H Gacaferi, A Wiberg, G Bond-Smith, E Gemmill, E Lenzi, D Sapre, P Herrod, H Boyd-Carson, G Garcea, E Issa, A Jackson, T Fashina, H Pan, B Farquharson, H Shafiq, O Emanuel, S Mahdi, S Jeyarajah, L Finch, G Whiting, L Pigott, J Martin, AK Siriwardena, K Beatson, L Abawi, W Lam, W Rea, B Andrews, B Al-Sarireh, F Soliman, J Burridge, C Jenvey, M Hammoda, M Hollyman, L Merker, J Richards, V Sukumaran, S Rogers, C Payne, S Bibi, K Raza, N Ul Ain, S Dronamraju, S Patil, S Nachimuthu, S Ravindran, S Patel, B Ivanov, M Patel, F Ejtehadi, J Jebamani, MM Akhter Rahman, H Woodun, A De Prendergast, A Afzal, E Bota, A Gupta, SR Abdul, R Karmarkar, E Crockett, L Evans, B Appleton, E Griffiths, O Dada, R Kulkarni, H Albirnawi, P Gravestock, C Vincenti, S Taribagil, B Dent, C Tse, B Clayton, E Burdekin, L Bannister, I Alam, J Gray, M Mactier, A Pollock, V Gough, SR Kanchustambam, M Ridgway, K Arujunan, S Gopalswamy, J Monteiro De Barros, T Lyons, D Griffith, AK Awan, J Latif, N Bandlamudi, I Bhatti, DA Raptis, N Machairas, T Pissanou, J Mestre-Costa, C Hidalgo Salinas, JM Pollok, M Al-Ardah, A White, E Watson-Jones, T Rontree-Carey, T Boyce, P Hawkin, A Elmaradny, K Ross, E Adu-Peprah, K Pinto, D Dunne, R Mccready, G Nita, P Szatmary, VL Tay, K Rajput, I Rajendran, M Chaudhury, G Zambas, C Swaminathan, QAA Atif, T Barrow, O Williams, A Malik, S Conroy, S Lindley, K Gilmore, E Boden, SK Richards, I Hraishawi, P Polak, D Mclaughlin, D Deeny, R Shuttleworth, A Harris, A Peilober-Richardson, GC Morris, X Sara, H Almourad, Y Ang, R Smyth, D Ding, J Foster, A Bond, Y Kumar, A Ahmad, D Radoi, A Alkaili-Alyamani, S Balakrishnan, RY Satchidanand, AS Danwaththa Liyanage, I Blake, M Ransome, C Weerasinghe, C Kenington, K Mayo, M Mohammed, AJ Cockbain, A Peckham-Cooper, G Mccauley, C Gordon, A Smith, W Hawkins, S Chakravartty, C Baillie, R Kenny, A Kumar, G Koimtzis, E Bellamy, A Menon, A Kanakala, EJ Nevins, A Madhavan, S Thulasiraman, K France, A O’Connor, D Idama, C Raslan, S Sridhar, M Parveen, T Mubashar, S Jarvis, I Cakmak, C Wright, S Andrews, Y Abdelsaid K Abdul Aal, B Jayasankar, J Morilla, M Shehata, N Subba, N Tewari, C El-Sayed, D Somaie, N Beheiry, E Douka, S Arumugam, I Wijetunga, E Leivers, B Ibrahim, K Khan, J Wheat, J Christopher, R Barnett, H Elberm, J Booker, S Ashai, D Berry, A Luhmann, A Sgro, MM Rashid, M Galea, J Jeyakumar, P Marriott, S Zafar, A Baker, D Yershov, G Galanopoulos, A Gupta, R Jordan, C Peinado Garcia, N Anyaugo, MF Bath, J Evans, J Omatseye, L Roberts, EO Argyriou, M Machesney, C Parmar, S Clark, H Khalil, S Unsworth, M Mlotshwa, N Ayoub, A Aboelkhair, E Iosif, N Mohamed, E Reynolds, E Mackender, D Robinson, W Mufti, K Fischkoff, N Coleman, S Anantha Sathyanarayana, G Deutsch, M Giangola, D Lin, M Weiss, C Chung, A Nguyen, J Mueller, M Dabit, J Gordon, E McGuire, O Rashid, E Georgi, M Gallo, JW Kunstman, NV Peters, R O’Connor, B Bhattacharya, E Onkendi, AP Santos, R Richmond, M Warren, K Zhang, R Broderick, B Clary, S Horgan, J Doucet, A Liepert, L Harmon, C McCall, JG Sham, E Williams, KP Labadie, NM Clark, LK Dickerson, CW Hammill, G Williams, B Kushner, H Cos, J Zarate Rodriguez, K Bailey, IMN Al-Raimi, K Al-Zazay, S Ahmed Mohammed Al-Mahdi, S Mohammed Aldowbli, M Al-Shehari, S Shream, S Al-Ameri, M Aeed, H Al-Naggar, M Aldawbali, R Alsayadi, M Alsayadi

**Affiliations:** Regional Hepato-Pancreato-Biliary Unit, Manchester Royal Infirmary, Manchester, UK; Cambridge Hepato-pancreato-biliary Unit, Addenbrooke's Hospital, Cambridge, UK

## Abstract

**Background:**

This study provides a global overview of the management of patients with acute cholecystitis during the initial phase of the COVID-19 pandemic.

**Methods:**

CHOLECOVID is an international, multicentre, observational comparative study of patients admitted to hospital with acute cholecystitis during the COVID-19 pandemic. Data on management were collected for a 2-month study interval coincident with the WHO declaration of the SARS-CoV-2 pandemic and compared with an equivalent pre-pandemic time interval. Mediation analysis examined the influence of SARS-COV-2 infection on 30-day mortality.

**Results:**

This study collected data on 9783 patients with acute cholecystitis admitted to 247 hospitals across the world. The pandemic was associated with reduced availability of surgical workforce and operating facilities globally, a significant shift to worse severity of disease, and increased use of conservative management. There was a reduction (both absolute and proportionate) in the number of patients undergoing cholecystectomy from 3095 patients (56.2 per cent) pre-pandemic to 1998 patients (46.2 per cent) during the pandemic but there was no difference in 30-day all-cause mortality after cholecystectomy comparing the pre-pandemic interval with the pandemic (13 patients (0.4 per cent) pre-pandemic to 13 patients (0.6 per cent) pandemic; *P* = 0.355). In mediation analysis, an admission with acute cholecystitis during the pandemic was associated with a non-significant increased risk of death (OR 1.29, 95 per cent c.i. 0.93 to 1.79, *P* = 0.121).

**Conclusion:**

CHOLECOVID provides a unique overview of the treatment of patients with cholecystitis across the globe during the first months of the SARS-CoV-2 pandemic. The study highlights the need for system resilience in retention of elective surgical activity. Cholecystectomy was associated with a low risk of mortality and deferral of treatment results in an increase in avoidable morbidity that represents the non-COVID cost of this pandemic.

## Teaser

CHOLECOVID is a global overview of the management of patients with acute cholecystitis during the first 2 months of the SARS-CoV-2 pandemic. Data are compared with an equivalent pre-pandemic time interval.

## Introduction

Acute cholecystitis is inflammation of the gallbladder, typically due to gallstones^1^. Management of patients with this condition is a major global healthcare burden^2^. International guidelines provide information on standards for diagnosis and treatment^3,4^. In patients without major co-morbidity, laparoscopic cholecystectomy during index admission is the recommended treatment for acute cholecystitis^3–5^. Globally, laparoscopic cholecystectomy is among the most frequently undertaken general surgical procedures with an estimated 115 performed per 100 000 of the world's population every year.^6^

In those who are unfit for surgery, treatment with antibiotics may be used as a temporizing option or as an attempt to control symptoms^1^. Radiologically guided percutaneous cholecystostomy can also be used in patients who are unfit for surgery or if operative treatment is not feasible^4,7,8^.

The outbreak due to the novel coronavirus, severe acute respiratory distress syndrome coronavirus 2 (SARS-CoV-2), presented a significant challenge to healthcare systems across the world^9^. With the WHO declaration of a global pandemic on 12 March 2020, healthcare resources were re-directed to care for patients with COVID-19^10^. Subsequently, healthcare organizations across the world published guidelines for the management of cholecystitis during the pandemic^11–14^. Patients requiring elective cholecystectomy had their treatment deferred where possible, with only those with the most urgent presentations undergoing surgery. In parallel there was emerging evidence of high rates of postoperative pulmonary complications in patients with perioperative SARS-CoV-2 infection^15^. This led to recommendations that thresholds for surgery during the COVID-19 pandemic should be higher and that consideration should be given to postponing elective cholecystectomy together with utilization of non-operative treatment for acute cholecystitis.

Although deferral or avoidance of treatment for acute cholecystitis was thus necessary during the initial stages of the SARS-CoV-2 pandemic, the consequences for these patients are not well understood. The present study examines global changes in the management and clinical outcomes of patients with acute cholecystitis during the SARS-CoV-2 pandemic. The CHOLECOVID study places emphasis on how management was changed during the pandemic, the consequences of these changes, and seeks to identify themes which may help clinicians address the management of acute cholecystitis in the ongoing pandemic setting.

## Methods

### Design

CHOLECOVID is an international, multicentre, observational comparative study of patients admitted to hospital with acute cholecystitis during the COVID-19 pandemic. Data on presentation and outcome were collected for a 2-month study interval coincident with the WHO declaration of the SARS-CoV-2 pandemic. These data are compared with a 2-month time interval from before the pandemic.

### Setting

The study was open to any hospital globally that managed patients with acute cholecystitis.

### Participants

Data were collected retrospectively for patients admitted with acute cholecystitis at participating centres in two 8-week time intervals. The index time interval was the 2 months from the WHO declaration of a SARS-CoV-2 pandemic (12 March 2020 to 12 May 2020). The comparator time interval was the 2 months from 12 September 2019 to 12 November 2019 before the pandemic. Consecutive adult patients (older than 18 years) with a clinical diagnosis of acute cholecystitis constituted the study population.

### Definitions of acute cholecystitis and SARS-COV-2 used in this study

Acute cholecystitis was defined according to the Tokyo criteria (2018), with at least one local sign of inflammation (Murphy's sign positive or right upper quadrant mass/pain/tenderness), at least one systemic sign of inflammation (fever or elevated C-reactive peptide (CRP) or elevated white cell count, and imaging findings characteristic of acute cholecystitis by at least one imaging modality (transabdominal ultrasonography, CT or magnetic resonance cholangiopancreatography (MRCP))^1^.

SARS-CoV-2 infection in the peri-admission interval (positivity 7 days before admission to 30 days from day of admission) was defined as a positive quantitative reverse transcriptase PCR (RT­–PCR) test, including nasopharyngeal swab or bronchoalveolar lavage (BAL) performed according to local protocols. As quantitative RT–PCR testing was not available at all participating hospitals in the early stages of the COVID-19 pandemic, patients were also classed as positive if they had a radiological (chest X-ray or CT thorax) or a clinical diagnosis (cough, fever, and loss of sense of smell/taste) of COVID-19^16^.

### Study data set

Descriptive data are provided on global patient cohorts from the pre-pandemic and pandemic time intervals.

#### Participating site profiles

Each participating site completed a site profile questionnaire for each data collection interval to describe local processes and resource provision. This specifically assessed the number of consultants/attending surgeons routinely performing cholecystectomy and the availability of daily urgent dedicated operating lists for cholecystectomy (termed ‘hot’ lists in some healthcare systems), together with operating list availability for cholecystectomy during the index admission with acute cholecystitis. In addition, the questionnaire assessed onsite interventional radiology and endoscopic retrograde cholangiopancreatography (ERCP) provision. Records with more than 5 per cent incomplete variables were excluded from the analyses. Hospitals submitting data to the study were included in analyses if at least one eligible patient record was uploaded per time interval. International health settings were categorized as either low/middle-income country (LMIC) or high-income country (HIC) according to the Human Development Index (HDI) of the United Nations Development programme^17^.

#### Demographic data and diagnosis of acute cholecystitis

Demographic variables collected included age at time of admission, sex, BMI, and Charlson co-morbidity index (CCI)^18^. Admission blood tests were recorded including leucocyte count (WCC), CRP, haemoglobin, platelet count, and enzymatic liver function tests. Acute kidney injury was also recorded where present, graded according to Kidney Disease Improving Global Outcomes guidance^19^. Data were collected for imaging modality utilized and timing of these tests following admission. Data were also collected for Tokyo grade of acute cholecystitis, use of critical care support on admission, and SARS-COV-2 status.

#### Management and outcome of acute cholecystitis

Data were collected on management of acute cholecystitis including non-operative (conservative) management, the use of cholecystostomy, and cholecystectomy. SARS-CoV-2 infection in the peri-admission interval is reported for the pandemic cohort. Duration of hospital stay, 30-day re-admission rates, and 30-day all-cause mortality after conservative management, cholecystostomy and cholecystectomy are provided. Data were collected on complication profiles after laparoscopic cholecystectomy.

#### Global overview of the management of acute cholecystitis

Global patterns in the management of acute cholecystitis are reported with further analysis by continent and HDI.

### Data collection procedure

Data collection followed a previously validated collaborative research methodology^15^. Anonymized data were submitted and stored online via the Research Electronic Data Capture (REDCap) web application, hosted on secure servers at the University of Manchester (Manchester, UK).

### Statistical analysis

Continuous data were assessed for distribution, summarized as mean (s.d.) or median (interquartile range; i.q.r.) with appropriate parametric or non-parametric tests performed. Categorical data were expressed as counts and percentages with differences tested with chi-squared or Fisher's exact tests.

Multiple logistic regression was used to investigate mortality from cholecystitis during the two time intervals. Covariates included in the model were age, sex, CCI^18^, WCC, acute kidney injury^19^, and Tokyo grade of cholecystitis^1^. Hospitals nested within country were added as random effects and final model selection was performed by minimizing the Akaike information criterion and maximizing the *c* statistic. After adjustment for case mix, variation in intervention for acute cholecystitis between the pre-pandemic and pandemic intervals was explored with Bland­–Altman plots^20^.

The influence of SARS-COV-2 infection on 30-day mortality was explored through mediation analysis with a natural-effects model^21^. The total effect underwent two-way decomposition into the effect of the time interval of surgery (direct effect) and the effect mediated by SARS-CoV-2 infection (indirect effect). This model was based around an *a priori* hypothesis that SARS-CoV-2 infection would significantly increase the 30-day mortality.

For all analyses, the threshold of two-sided statistical significance was set at *P* < 0.05. Effect estimates were summarized as OR with 95 per cent confidence interval (c.i.). All analyses were performed with R 3.6.1 (R Foundation for Statistical Computing, Vienna, Austria).

### Ethics

Each study centre was required to comply with their appropriate institutional research board or audit committee and a statement confirming that this was in place was required before data acceptance. For the UK's National Health Service, the National Research Ethics Service decision tool (http://www.hra-decisiontools.org.uk/research/) confirmed that the study did not require research registration.

## Results

### Overview

**Fig. 1:**
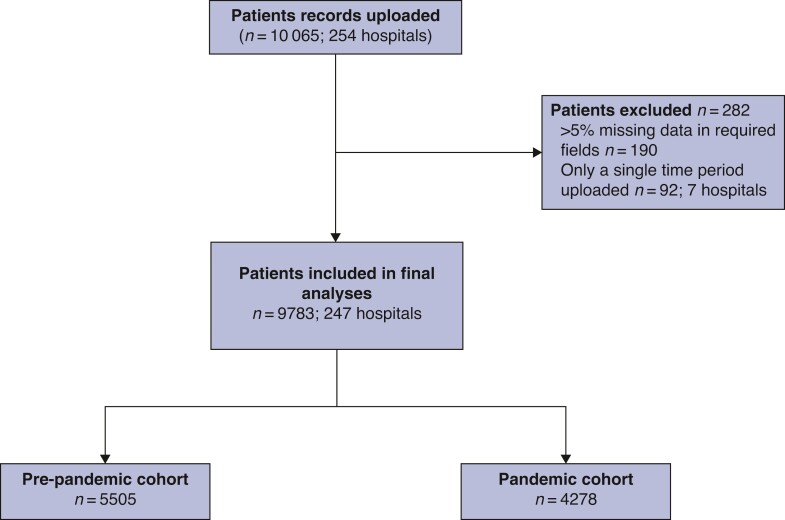
**Flow chart of patients in CHOLECOVID study**.

This study collected data on 10 065 patients with acute cholecystitis admitted to 254 hospitals during the two study intervals (*Fig. 1* ). After exclusions for incomplete data, 9783 patients from 247 hospitals from 40 countries constitute the study population. Of these, 5505 (56.3 per cent) were admitted with acute cholecystitis before the COVID-19 pandemic (the pre-pandemic cohort), and 4278 (43.7 per cent) were admitted during the pandemic (pandemic cohort). Data from 60 hospitals (24.3 per cent) and 1743 patients (17.8 per cent) were from LMICs; the remaining 187 (75.7 per cent) hospitals and 8040 patients (82.2 per cent) were from HICs. In terms of type of healthcare facility, 97 (39.3 per cent) were secondary care centres, and 150 (60.7 per cent) were tertiary care centres.

### Profile of participating sites

Fewer sites (38 per cent compared with 45.4 per cent, *P* = 0.117) had urgent cholecystectomy list availability during the pandemic, with an increase in the number of sites providing either no sessions or more than one session per week (*P* = 0.002) (*Table 1*). There was no significant difference in the availability of either onsite interventional radiology or ERCP services.

**Table 1:** Profile of participating clinical sites

	Pre-pandemic	Pandemic	*P*
**Total number of sites**	242	242	NA
Number of consultants/attendings routinely performing cholecystectomy			
1	6 (2.5)	11 (4.5)	0.069
2	11 (4.5)	16 (6.6)	
3	8 (3.3)	15 (6.2)	
4	27 (11.2)	32 (13.2)	
5	13 (5.4)	22 (9.1)	
≥6	177 (71.7)	146 (60.3)	
**Daily urgent gallbladder operating list**	110 (45.5)	92 (38.0)	0.117
Urgent gallbladder list frequency			
No sessions	132 (54.5)	150 (62.0)	0.002
Less than 1 session per week	7 (2.9)	18 (7.4)	
1–2 sessions per week	29 (12.0)	33 (13.6)	
3–4 sessions per week	35 (14.5)	23 (9.5)	
5 or more sessions per week	39 (16.1)	18 (7.4)	
**Onsite interventional radiology**	206 (85.1)	201 (83.1)	0.619
**Onsite ERCP service**	214 (88.4)	208 (86.0)	0.496

Participating site details are available for 242 hospitals. Values in parentheses are percentages. NA, not available; ERCP, endoscopic retrograde cholangiopancreatography.

### Characteristics of patients with acute cholecystitis

There was no significant difference in age, BMI, or CCI between the pre-pandemic and pandemic cohorts (*Table 2*). During the pandemic there was a significant shift in severity of acute cholecystitis with more grade II (1653 patients (30 per cent) pre-pandemic compared with 1499 patients (35 per cent) pandemic) and more grade III disease (208 patients (3.8 per cent pre-pandemic) compared with 175 patients (4.1 per cent pandemic); *P* < 0.001).

**Table 2:** Baseline characteristics of patients with cholecystitis

	Pre-pandemic	Pandemic	*P*
**Total number of patients**	5505	4278	NA
Age, years			
Median (i.q.r.)	60.0 (45.0–74.0)	60.0 (44.0– 74.0)	0.865
Sex			
Male (%)	2456 (44.6)	2011 (47.0)	0.019
BMI (%)			
<18.5	64 (1.2)	57 (1.3)	0.088
18.5–24.9	1251 (22.7)	890 (20.8)	
25–29.9	1559 (28.3)	1238 (28.9)	
30–39.9	1064 (19.3)	907 (21.2)	
≥40	218 (4.0)	164 (3.8)	
Unknown	1349 (24.5)	1022 (23.9)	NA
Charlson co-morbidity index			
Median (i.q.r.)	2.0 (0–4.0)	2.0 (0–4.0)	0.948
White cell count			
Mean(s.d.)	12.4(5.3)	12.9(5.6)	<0.001
C-reactive peptide			
Median (i.q.r.)	39.3 (8.6–145.4)	39.0 (7.8–160.0)	0.908
ALP			
Median (i.q.r.)	100.0 (73.0–161.0)	101.0 (74.0–170.0)	0.294
ALT			
Median (i.q.r.)	31.0 (18.0–91.8)	36.0 (20.0–102.0)	<0.001
Bilirubin			
Median (i.q.r.)	15.0 (8.0–27.0)	15.0 (8.3–28.0)	0.019
Acute kidney injury (%)			
None	5011 (91.0)	3795 (88.7)	0.001
Stage I	336 (6.1)	330 (7.7)	
Stage II/III	158 (2.9)	153 (3.6)	
Tokyo severity grade (%)			
Grade I (mild)	3644 (66.2)	2604 (60.9)	<0.001
Grade II (moderate)	1653 (30.0)	1499 (35.0)	
Grade III (severe)	208 (3.8)	175 (4.1)	
Critical care on admission (%)			
No	5381 (97.7)	4207 (98.3)	0.045
Yes	124 (2.3)	71 (1.7)	
**SARS-CoV-2 (pandemic interval only)**			
**Total SARS-CoV-2 positivity (%)**			197 (4.5)
SARS-COV-2 infection 7 days before admission (%)			
Positive			35 (0.8)
Negative			1093 (25.6)
Unknown			3150 (73.6)
Method of diagnosis pre-admission (%)			
Nasopharyngeal swab			33 (94.3)
X-ray			9 (25.7)
CT			6 (17.1)
Clinical diagnosis			11 (31.4)
SARS-COV-2 infection during admission (%)			
Positive			180 (4.2)
Negative			3179 (74.3)
Unknown			919 (21.5)
Positive tests during admission (% of cohort)			
Nasopharyngeal swab			127 (2.9)
X-ray			47 (1.1)
CT			57 (1.3)
Clinical diagnosis			78 (1.8)

Values in parentheses are percentages unless otherwise specified. ALP, alkaline phosphatase; ALT, alanine transaminase; i.q.r., interquartile range; NA, not available. Note that the total number of SARS-Cov-2 positive results of 197 includes patients who tested positive in the 7 days before surgery in addition to patients diagnosed as inpatients. Eighteen patients who tested positive in the 7 days before admission were also positive on admission tests. Note also that more than one method of diagnosis of SARS-Cov-2 was reported thus patients could have a combination of nasopharyngeal swab, X-ray, CT, and clinical diagnosis.

Fewer patients were admitted directly to critical care during the pandemic (71 patients (1.7 per cent) *versus* 124 patients (2.3 per cent), *P* = 0.045). Of patients admitted during the pandemic, 197 patients (4.5 per cent) tested positive for SARS-CoV-2 infection of whom 35 patients (0.8 per cent) tested positive in the 7-day interval before admission, with 94.3 per cent of these diagnosed by nasopharyngeal swab.

### Diagnosis of acute cholecystitis

There was reduced utilization of transabdominal ultrasonography during the pandemic (82.2 per cent pre-pandemic *versus* 73.7 per cent pandemic; *P* < 0.001), with an increase in CT imaging (36.6 per cent pre-pandemic *versus* 46.2 per cent pandemic; *P* < 0.001) (*Table 3*). The use of CT as a first-line imaging modality increased during the pandemic with a concomitant reduction in first-line transabdominal ultrasonography. The delay from admission to ultrasound scan was shorter during the pandemic (pre-pandemic mean(s.d.) 0.8(3.2) days *versus* pandemic 0.6(1.6) days; *P* < 0.001).

**Table 3:** Diagnosis of acute cholecystitis

	Pre-pandemic	Pandemic	Total	*P*
**Total number performed (% of patients in cohort)**				
Ultrasound	4525 (82.2)	3152 (73.7)	7677 (78.5)	<0.001
CT	2014 (36.6)	1976 (46.2)	3990 (40.7)	<0.001
MRCP	858 (15.6)	714 (16.7)	1572 (16.1)	0.179
**First-line imaging modality utilized (%)**				
US	4013 (72.9)	2742 (64.1)	6755 (69.0)	<0.001
CT	1409 (25.6)	1450 (33.9)	2859 (29.3)	
MRCP	83 (1.5)	86 (2.0)	169 (1.7)	
**Mean (s.d.) timing of imaging modality in days**				
Ultrasound	0.8 (3.2)	0.6 (1.6)	0.7 (2.7)	0.001
CT	0.9 (2.7)	0.8 (2.5)	0.9 (2.6)	0.153
MRCP	3.4 (3.9)	2.7 (3.1)	3.1 (3.6)	<0.001

MRCP, magnetic resonance cholangiopancreatography; US, transabdominal ultrasound scan.

Note that more than one diagnostic test could be used.

### Management of acute cholecystitis

There was a significant increase in the proportion of patients with grade II or grade III cholecystitis being managed conservatively during the pandemic with fewer undergoing cholecystectomy (960 undergoing cholecystectomy pre-pandemic compared with 720 during the pandemic; *P* < 0.001) (*Table 4*). Forty-six patients who were positive for SARS-CoV-2 underwent cholecystectomy during the pandemic interval.

**Table 4:** Management and overall outcome of acute cholecystitis

	Pre-pandamic				Pandemic			
	Conservative management	Cholecystostomy	Cholecystectomy	*P*	Conservative management	Cholecystostomy	Cholecystectomy	*P*
**Tokyo severity grade (per cent)**								
Grade I (mild)	1430 (68.2)	89 (27.6)	2125 (68.9)	<0.001	1231 (64.5)	95 (25.7)	1278 (63.9)	<0.001
Grade II (moderate)	598 (28.5)	171 (53.1)	885 (28.7)		612 (32.0)	216 (58.4)	671 (33.6)	
Grade III (severe)	70 (3.3)	62 (19.3)	75 (2.4)		67 (3.5)	59 (15.9)	49 (2.5)	
**SARS-CoV-2 infection in the peri-admission interval (per cent)**								
Positive	NA	NA	NA	NA	107 (5.6)	44 (11.9)	46 (2.3)	<0.001
Negative	NA	NA	NA	NA	1803 (94.4)	326 (88.1)	1952 (97.7)	
**Median (i.q.r.) duration of hospital stay, days**	5.0 (3.0–8.0)	11.0 (7.0–17.0)	4.0 (3.0–6.0)	<0.001	5.0 (3.0–7.0)	9.0 (6.0–15.0)	4.0 (2.0–6.0)	<0.001
**30-Day re-admission (per cent)**	268 (12.8)	121 (37.6)	223 (7.2)	<0.001	227 (11.9)	143 (38.6)	137 (6.9)	<0.001
**30-day all-cause mortality (per cent)**	60 (2.9)	31 (9.6)	13 (0.4)	<0.001	63 (3.3)	33 (8.9)	13 (0.6)	<0.001

Conservative management, cholecystostomy, and cholecystectomy before and during the pandemic are compared. i.q.r., interquartile range; NA, not available.

Median (i.q.r.) duration of hospital of stay was greatest after cholecystostomy (11 (7–17) days before the pandemic and 9 (6–15) days during the pandemic), with no change in inpatient stay after cholecystectomy before or during the pandemic (4 (3–6) days and 4 (2–6) days) respectively.

There was a reduction (both absolute and proportionate) in the number of patients undergoing cholecystectomy from 3095 patients (56.2 per cent) pre-pandemic to 1998 patients (46.2 per cent) during the pandemic but there was no difference in 30-day all-cause mortality after cholecystectomy comparing the pre-pandemic interval to the pandemic (13 patients (0.4 per cent) pre-pandemic to 13 patients (0.6 per cent) pandemic; *P* = 0.355).

### Complication profiles of cholecystostomy and cholecystectomy

#### Cholecystostomy

Overall, 119 (17.2 per cent) of patients experienced procedure-related complications from cholecystostomy (*Table 5*). There was no difference in the frequency of complications before and during the pandemic. Re-intervention was required in 162 cases (23.4 per cent). Once placed, 78.9 per cent of patients were discharged home with a cholecystostomy tube *in situ*. Median duration of hospital stay for patients with cholecystitis was longest for the group undergoing cholecystostomy (*Table 4*).

**Table 5:** Complication profiles and peri-procedural outcomes of cholecystostomy and cholecystectomy

	Pre-pandemic	Pandemic	Total	*P*
**Cholecystostomy**				
Total (*n*)	322	370	692	NA
Complication	51 (15.8)	68 (18.4)	119 (17.2)	0.434
Bleeding	9 (2.8)	12 (3.2)	21 (3.0)	0.904
Bile leak	5 (1.6)	9 (2.4)	14 (2.0)	0.583
Intra-abdominal collection	6 (1.9)	6 (1.6)	12 (1.7)	0.990
Occlusion	7 (2.2)	12 (3.2)	19 (2.7)	0.532
Dislodgement	27 (8.4)	37 (10.0)	64 (9.2)	0.549
Perforation	1 (0.3)	1 (0.3)	2 (0.3)	0.990
Re-intervention	79 (24.6)	83 (22.4)	162 (23.4)	0.559
Discharged with cholecystostomy *in situ*	248 (77.0)	298 (80.5)	546 (78.9)	0.299
Pulmonary complication	34 (10.6)	57 (15.4)	91 (13.2)	0.077
Pulmonary complication (by SARS-CoV-2)				
Negative	NA	39 (10.5)	NA	NA
Positive	NA	18 (4.9)	NA	
Pneumonia	22 (6.8)	38 (10.3)	60 (8.7)	0.142
ARDS	8 (2.5)	13 (3.5)	21 (3.0)	0.572
Unexpected ventilation	10 (3.1)	12 (3.2)	22 (3.2)	0.990
Pulmonary embolus	2 (0.6)	2 (0.5)	4 (0.6)	0.990
**Cholecystectomy**				
Total (*n*)	3085	1998	5083	NA
Clavien–Dindo grade				
Minor (I–II)	434 (14.0)	370 (18.5)	804 (15.8)	<0.001
Major (III–V)	165 (5.3)	112 (5.6)	277 (5.4)	
30-Day postoperative mortality	13 (0.4)	13 (0.6)	26 (0.5)	0.355
Post-cholecystectomy biliary complications				
Bile leak	85 (2.7)	54 (2.7)	139 (2.7)	0.127
Bile duct injury	12 (0.4)	7 (0.4)	19 (0.4)	0.651
Pulmonary Complications	85 (2.7)	54 (2.7)	139 (2.7)	0.992
Pulmonary complications (by SARS-CoV-2)				
Yes (negative)	NA	42 (2.1)	NA	NA
Yes (positive)	NA	12 (0.6)	NA	
Pneumonia	46 (1.5)	28 (1.4)	74 (1.5)	0.896
ARDS	12 (0.4)	13 (0.6)	25 (0.5)	0.270
Unexpected ventilation	27 (0.9)	10 (0.5)	37 (0.7)	0.174
Pulmonary embolus	4 (0.1)	4 (0.2)	8 (0.2)	0.794

Figures in parentheses represent percentages. ARDS, adult respiratory distress syndrome; i.q.r., interquartile range; NA, not available. Unexpected ventilation refers to unplanned urgent (emergency) intubation. Clavien–Dindo grades relate to all complications pre-pandemic compared with post-pandemic.

#### Cholecystectomy

Cholecystectomy complications were assessed by Clavien–Dindo grade. Although there were significantly more complications after cholecystectomy during the pandemic interval, this was mainly due to differences in minor complications (Clavien–Dindo grade I–II, 14.0 per cent pre-pandemic *versus* 18.5 per cent during pandemic; Clavien–Dindo grade III–V, 5.3 per cent pre-pandemic *versus* 5.6 per cent during pandemic; *P* < 0.001). There was no difference in the post-cholecystectomy complications of bile leak, bile duct injury, or overall pulmonary complications.

### Management of cholecystitis comparing HICs with LMICs

There was a significant increase in conservative management of acute cholecystitis during the pandemic. In HICs this was from 1915 patients (42.9 per cent) to 1743 patients (48.7 per cent) (*P* < 0.001). In LMICs this was from 183 patients (17.5 per cent) to 167 patients (24.0 per cent) (*P* < 0.002). Proportionately more patients underwent conservative management in HICs in both time intervals (1915 patients (42.9 per cent) before the pandemic and 1743 patients (48.7 per cent) during the pandemic).

**Fig. 2:**
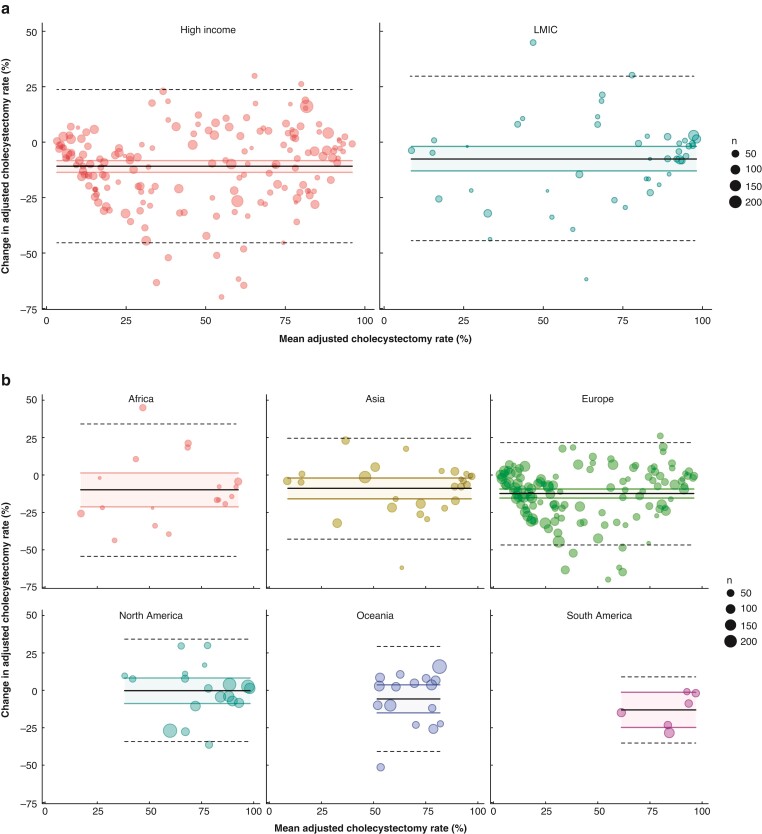
Bland–Altman plots of change in cholecystectomy rate during the pandemic compared with pre-pandemic

In relation to cholecystectomy, 2764 procedures (89.2 per cent) were undertaken laparoscopically before the pandemic and 1760 procedures (87.9 per cent) were undertaken during the pandemic. This difference was not significant (*P* = 0.173). The Bland–Altman plots (*Fig. 2*) show that HICs showed a negative difference in adjusted cholecystectomy rates compared with LMICs with the pandemic rate being lower than the pre-pandemic rate. By continent, the reduction in adjusted cholecystectomy rates was greatest for Europe, South America, and Oceania.

**a** Data stratified by high-income *versus* low/middle-income countries. **b** Data stratified by continent. Each plot is expressed as percentages of the difference in adjusted cholecystectomy rates on the *y* axis (pandemic rate − pre-pandemic rate) *versus* the mean of the two rates across the two intervals ((pandemic rate + pre-pandemic rate)/2). Mean change is represented as a solid black line with 95 per cent confidence intervals for the mean represented as shaded areas. The limits of agreement, represented as dashed black lines, are defined as the mean difference with 1.96 s.d. of differences.

#### Effect of SARS-CoV-2

**Fig. 3:**
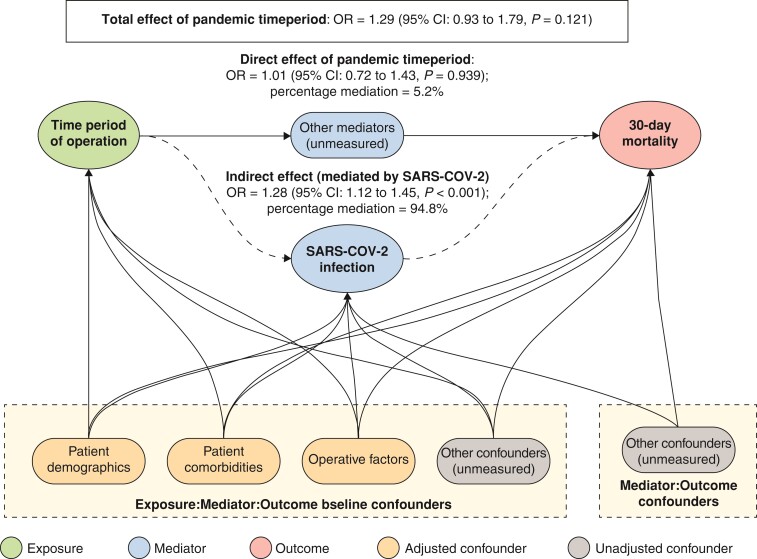
Mediation analysis of excess odds of death in patients with acute cholecystitis during the SARS-CoV-2 pandemic by SARS-CoV-2 infection

Following adjustment with a natural-effects mediation analysis, an admission with acute cholecystitis during the pandemic was associated with a non-significant increased risk of death (OR 1.29, 95 per cent c.i. 0.93 to 1.79, *P* = 0.121) (*Fig. 3*). In the absence of SARS-CoV-2 infection, there was no difference in the mortality rate between the time intervals (OR 1.01, 95 per cent c.i. 0.72 to 1.43, *P* = 0.939). The indirect effect of excess odds of death due to SARS-CoV-2 infection was a significant increase in 30-day mortality (OR 1.28, 95 per cent c.i. 1.12 to 1.45, *P* < 0.001).

The model used patient and disease characteristics and SARS-CoV-2 infection as covariates. The proportion of the total effect mediated by SARS-CoV-2 infection for mortality in patients with acute cholecystitis during the pandemic with bootstrapped 95 per cent confidence intervals is reported.

## Discussion

CHOLECOVID is the largest global observational cohort study to date comparing the management of patients with acute cholecystitis during the initial stages of the SARS-COV-2 pandemic with a similar time interval before this. Although studies have examined the effects of the pandemic on surgery in general and on specific aspects such as surgical oncology^22^, CHOLECOVID provides a unique perspective on the spectrum of outcomes for patients with acute cholecystitis.

What can be learnt from this large study of 9783 patients with acute cholecystitis admitted to 247 hospitals in 40 countries during the two study intervals? The surgical workforce was disrupted by the pandemic with a reduction in the number of centres with six or more consultants/attending surgeons performing cholecystectomy.

In parallel, there was a significant shift in the severity of acute cholecystitis. CHOLECOVID did not collect information on delay from onset of symptoms to admission, and declined or avoided hospital admission rates. However, it is possible to speculate that the shift in admission severity reflects at least in part, delayed presentation^23,24^ and/or increased difficulty (or avoidance) of hospital admission^25^. The reduction in admission directly to critical care is thought to represent re-prioritization of bed use with greater resource being diverted to care for patients with COVID-19^26^. Note that the 4.5 per cent (197 of 4278) positive rate for SARS-CoV-2 infection is likely to reflect scarcity of testing in the early stages of the pandemic. The shift in diagnostic modality for acute cholecystitis, away from transabdominal ultrasound and towards CT is possibly reflective of pressures to undertake social distancing and may relate to guidance on the use of thoracic CT in the early stages of the pandemic^27^.

In terms of management, there was a shift to a greater proportion of grade II and grade III cholecystitis patients being treated conservatively during the pandemic and fewer undergoing cholecystectomy. This finding is similar to the experience reported from a national overview study of 4035 patients undergoing cholecystectomy in Germany during the initial stages of the pandemic^28^.

Although cholecystostomy may provide a useful temporizing procedure, it was associated with a 17.2 per cent incidence of procedure-related complications; 23.4 per cent of patients required re-intervention and 78.9 per cent went home with the cholecystostomy tube *in situ*. While this may facilitate hospital discharge, it creates an additional burden of workload for community care.

In relation to cholecystectomy, there was no significant difference in the frequency of pulmonary complications, and although there was an increase in the overall complication profile this did not translate into increased 30-day mortality. Thus a key theme to emerge from CHOLECOVID is that while temporizing manoeuvres may get patients out of hospital quickly, the lack of provision of definitive treatment leads to ongoing problems.

The comparison of the management of cholecystitis in different parts of the world during the pandemic is interesting. Conservative management was more frequently employed in HICs before and during the pandemic. This may reflect availability of other treatment modalities such as interventional radiology facilities. Laparoscopic cholecystectomy was maintained throughout the pandemic. The Bland–Altman plot (*Fig. 2*) shows a greater change in adjusted cholecystectomy rates in HICs compared with LMICs. By continent, the greatest reductions were seen in Europe, Oceania, and South America and the least in North America, Africa, and Asia. It is important to avoid over-interpretation from these data, but the greater change in adjusted cholecystectomy rates in HICs may be a genuine finding due to the availability of other treatment options in these healthcare systems or may represent limitations in data capture. The role of socioeconomic status is also important as this can impact the use of cholecystectomy^29^.

Natural-effects mediation analysis showed that an admission with acute cholecystitis during the pandemic was associated with an overall non-significant increase in risk of death (OR 1.29, 95 per cent c.i. 0.93 to 1.79, *P* = 0.121) (*Fig. 3*).The indirect effect of excess odds of death due to SARS-CoV-2 infection was a significant increase in 30-day mortality (OR 1.28, 95 per cent c.i. 1.12 to 1.45, *P* < 0.001). Mediation analysis is routinely used to assess the effect of multiple factors on an outcome. Its limitations relate to the accuracy with which an individual contributing factor can be quantified.

When interpreting the findings, limitations of the study must be borne in mind. First, there was no independent validation of data. Thus, the data set could be influenced by acquisition bias and the allocated treatments influenced by allocation bias. Reporting could also have been influenced by sampling and attrition bias. However, previous data verification in national and international observational studies has shown acceptable accuracy in reporting when utilizing this method of data collection^6^. Second, the study interval was short and the pandemic interval encompassed the time of likely maximal disruption. Third, the choice of the early interval of the pandemic pre-dated mass population testing for SARS-Cov-2. Fourth, although CHOLECOVID was open to recruitment from centres across the globe there were more replies from HICs and from tertiary care facilities.

In summary, CHOLECOVID provides an overview of the treatment of patients with cholecystitis across the globe during the first months of the SARS-CoV-2 pandemic. The study shows that although healthcare priorities may need to reflect the pressing needs of patients with SAR-CoV-2, it must be appreciated that reallocation of resources occurs at a cost to patients with common surgical conditions, such as cholecystitis. The definitive and better outcomes from cholecystectomy shown here provide support for enhancing system resilience to retain activity during the ongoing pandemic. Deferral of treatment or change in treatment results in an increase in avoidable morbidity that represents the non-COVID cost of this pandemic.

## Supplementary Material

zrac052_Supplementary_Data

## Data Availability

Anonymized source data can be made available on request.

## Collaborators

### CHOLECOVID Collaborative

**Writing group**: Harry V M Spiers*, Omar Kouli*, Waheed U Ahmed, Rebecca Varley, Daniel Ahari, Leah Argus, Kenneth A McLean, Sivesh K Kamarajah, Peter Coe, Ewen A Griffiths, Anthony KC Chan, Christian Macutkiewicz, Saurabh Jamdar, Michael Wilson, Catherine Fullwood, Giles Toogood, Ajith K Siriwardena (senior author & overall guarantor).

**Joint first authors who contributed equally*

**Statistical analysis**: Omar Kouli, Kenneth McLean, Catherine Fullwood.

**Operations committee**: Daniel Ahari, Leah Argus, Rebecca Varley, Harry V M Spiers, Omar Kouli, Waheed Ahmed, Andrew Gilchrist, Matthew Goldsworthy, Majid Rashid.

**National Leads:** P Pockney (Australia), Varela J (El Salvador), Brindl N (Germany), Ramirez J (Guatemala), Marafante C (Italy), Iwao Y (Japan), Ghzawi A (Jordan), Elhadi M (Libya), Gacaferi H (The Netherlands), Varghese C (New Zealand), Adeyeye A (Nigeria), Alser O (Palestine), Teh C (Philippines), Prieto M (Spain), Hasan A (United Arab Emirates), Al-Naggar H (Yemen).

### Collaborators

*denotes principal investigator at each site.

**Argentina (AR):** Salgado R*, Veracierto F, Lancelotti T, Solinas D, Oddi R (Cemic (Centro De Educacion Medica E Investigaciones Clinicas)); Garcia FW*, Mazza Diez E, Andrade Ramirez MR, Bracco R, Fernandez D (Clinica Pueyrredon); Maraschio MA*, Obeide L, Giordano E, Alcaraz A, Marani MA (Hospital Privado Universitario De Cordoba); Aguirre N*, Luna F, Francesconi M, Chiham F, Ramos Cossio R (Petrona V. De Cordero); Alvarez FA*, Pantoja Pachajoa DA, Mandojana F (Clinica Universitaria Reina Fabiola); Merlo IG*, Gonzalez MH, Cervelo G, Puma R, Vardaro GF (Sanatorio San Justo).

**Australia (AU):** Davis A*, Jurat D, Guenoff C, Raubenheimer K,​​ Goddard K (Armadale Health Service); Brown K, Wegrecki KJ, Cheung HYC*, Yang M (Canterbury Hospital); Cheung H*, Siddiqui J, Ahn JH, Huynh R (Concord Repatriation General Hospital); Lam YH*, Afzal M, Ong BS, Chua MYM, Ly K, Thomson JE, Watson D (Flinders Medical Centre); Dawson AC*, Drane A, Van Ruyven S, Lun EWY (Gosford Hospital); Pockney P*, Ferguson M, Jeong JY, De Silva C, Wills V (John Hunter Hospital); Gundara J*, Mccourt E, Bong C, Tabone R, Wong WJ (Metro South Health (Redland/Logan Hospitals)); Gray A*, Koh D, Pollock M, Singhal S, Smith R (Monash Medical Centre); Dudi-Venkata NN*, Kanhere H, Stranz C, Seow W, Mansour LT, Wormald J (Royal Adelaide Hospital); Loveday BPT*, Thomson B, O’Donnell T, Milenkovski N (Royal Melbourne Hospital); Herath M*, Trochsler M, Farfus A, Maddern G, Bunjo Z, Kuan LL, Atanasov G (The Queen Elizabeth Hospital); Dawson A*, Drane A, van Ruyven S, Lun E (Wyong Public Hospital).

**Azerbaijan (AZ):** Samadov E*, Namazov I, Asgarov M, Ibrahimli A (Leyla Medical Center).

**Bahrain (BH):** Srinivasan M*, Saeed MF, Aljawder H, Juma I (King Hamad University Hospital).

**Brazil (BR):** Coimbra FJ*, Marques N (A.C. Camargo Cancer Center); Casteleins WA*, Petruzziello A, Jabur G, Rodriguez JFP, Buso PL (Hospital Universitario Cajuru).

**Canada (CA):** MacKenzie S*, Hsiao M, Sljivic I, Tecson A (Royal Columbian Hospital); Karanicolas PJ*, Roke R, Moon J, Butler EV (Sunnybrook Health Sciences Centre).

**Chile (CL):** Riquelme F*, Yanez M, Catan F, Uribe M (Hospital Del Salvador); Carriel F*, Oppliger F, Paredes A, Daroch D, Aguayo JC (Hospital Padre Hurtado).

**Colombia (CO):** Perez Rivera CJ*, Acosta Buitrago LM, Kadamani Abiyomaa A, Mosquera Paz MS, Cabrera P, Corso J (Fundacion Cardioinfantil - Instituto De Cardiologia).

**Cyprus (CY):** Ozcay N*, Ozant A, Arslan K, Besim H, Almezghwi H (Near East University Hospital).

**Egypt (EG):** Azzam AY* (Al-Azhar University Hospitals); Bessa S, El-Sayes I, Badawy A, Wael M (Alexandria University Medical Centre); El-Gendi A*, Azab MA (Cairo University Hospitals); Fayed M (El-Mahalla Hepatology Teaching Hospital); El Kassas M*, Gamal M, Tawheed A, Al Shafie A (Endemic Medicine Department,Helwan University); Emile S*, Elfallal A, Elfeki H, Shalaby M, Sakr A (Mansoura University Hospital); Elbahnasawy M*, Shama M, Abdel-Elsalam W, Abd-ElsalamS (Tanta University Hospital).

**El Salvador (SV):** Escobar Dominguez JE*, Medrano F, Gaitan S, Escalon Gonzalez OM (Central Military Hospital); Alfaro Varela JC*, Cea M, Interiano M (Instituto Salvadoreno Del Seguro Social (Salvadoran Institute Of Social Security)); Cabrera B* (Hospital Nacional San Juan de Dios).

**France (FR):** Lakkis Z*, Georges P, Antonot C, Magnin J (University Hospital Of Besançon).

**Germany (DE)**: Kamphues C*, Lauscher JC, Schineis C, Loch FN, Lee LD, Beyer K (Charite University Medicine, Campus Benjamin Franklin).

**Greece (GR):** Bouchagier K*, Galanis I, Bartziotas D (General Hospital Of Athens Evaggelismos); Lostoridis E*, Tourountzi P, Nagorni EA (Kavala General Hospital); Charalabopoulos A*, Baili E, Kyros E, Vagios I, Skotsimara A, Liakakos T, Alexandrou A, Papalampros A (Laiko General Hospital); Papadopolous V*, Tooulias A, Kentarchos I, Christou C, Tsoulfas G (Papageorgiou General Hospital).

**Guatemala (GT):** Tale-Rosales LF*, Lopez Muralles I, Melendez H, Bran G, Monroy Mahecha FA (General Hospital Dr. Juan Jose Arevalo Bermejo, Guatemalan Institute Of Social Security); Contreras JR*, Porras DE, Paiz E, Soto ER, Ixcayau Hernandez JR (Instituto Guatemalteco De Seguridad Social).

**India (IN):** Gupta A*, Rajput D, Kumar N, Mani R*, Kant R (All India Institute Of Medical Sciences Rishikesh); Sonkar AA*, Anand A, Agrawal MK, Gaurav K, Tripathi M (King Georges Medical University); Sikora S*, Bharathy K, Kumar Rangapa M, Khuller DS, K SK (Sakra World Hospital); Bhojwwani R*, Ayyar S, Jain N (Santokba Durlabhji Memorial Hospital); Mehraj A*, Hussain F, Nazir I, Shah M, Chowdri NA (Sher I Kashmir Institute Of Medical Sciences Srinagar).

**Iraq (IQ):** Hilmi A* (Ibn Sina Hospital).

**Italy (IT):** Argenio G*, Atelli P, Palladino E, Armellino MF (Aou San Giovanni Di Dio E Ruggi Daragona); Tamini N*, Nespoli LC, Degrate L, Angrisani M, Carissimi F, Bordoni P (ASST Monza - Ospedale San Gerardo); Fleres F*, Bordoni P, Clarizia G, Spolini A, Franzini M (General Surgery Unit,Ospedale Civile di Sondrio, ASST Valtellina E Alto Lario, Sondrio, Italy); Cucinotta E*, Badessi G, Mazzeo C, Viscosi F, Pintabona G (General and Emergency Surgery Unit, Azienda Ospedaliera Policlinico Universitario G. Martino, University of Messina, Messina, Italy); Campagnaro T*, Poletto E, Turri G, Ruzzenente A, Conci S, Guglielmi A (Azienda Ospedaliera Universitaria Integrata Verona); Feo C*, Fabbri N, Fazzin M, Giaccari S, Feo CV (Azienda Usl Di Ferrara); Massani M, Pelizzo P, Colella M, Tutino R* (Chirurgia 1 - Azienda ULSS2 Marca Trevigiana - Ospedale Regionale Treviso); Cappellacci F*, Medas F, Canu GL, Erdas E, Calò PG (Chirurgia Generale Polispecialistica Aou); Porcu A*, Perra T, Scanu AM, Feo CF, Fancellu A (Clinica Chirurgica Azienda Ospedaliero Universitaria (Aou) - Sassari); Germani P*, Giunta C, Biloslavo A, Abdallah H, Aizza G (Clinica Chirurgica Cattinara University Hospital); Barberis A*, Belli F, Santoliquido M, Filauro M (E.o. Ospedali Galliera, Genoa); Canonico G*, Nelli T, Di Martino C, Capezzuoli L, Anastasi A, Bressan L, Cortinovis S, Nagliati C (Hospital San Giovanni Di Dio); Colombo F*, Ferrario L, Bondurri A, Guerci C, Maffioli A (L. Sacco University Hospital); Catena F*, Perrone G, Giuffrida M, Morini A, Annicchiarico A (Maggiore Hospital); Gallo G*, Carpino A, Ferrari F, De Paola G, Sammarco G (Operative Unit Of Digestive Surgery, University “Magna Graecia” of Catanzaro, Catanzaro, Italy); Callari C*, Licari L, Sorce V, Di Miceli D (Ospedale Buccheri La Ferla Hospital); Lovisetto F*, Zonta S, Lovisetto F (Ospedale Castelli Verbania); Chessa A*, Fiorini A (Ospedale Civile San Giovanni Di Dio); De Manzoni Garberini A*, Angelini E (Ospedale Civile Spirito Santo); Marafante C*, Moggia E, Murgese A, Mungo S, Birolo SL, Garino M (Ospedale degli Infermi, Rivoli); Pipitone Federico NS*, Muratore A, Lunghi EG, Calabro M (Ospedale Edoardo Agnelli); Cianci P*, Enrico R, Capuzzolo S, Cafagna L, Minafra M (Ospedale Lorenzo Bonomo Chirurgia); Sasia D*, Gattolin A, Migliore M, Rimonda R, Travaglio E (Regina Montis Regalis Hospital, Mondovì); De Marco G*, Elter C, Bargellini T, D’amico S, Zambonin D, Caponi A (Ospedale San Giuseppe Di Empoli Chirurgia Generale); Calini G*, Puggioni A, Bresadola V (University Hospital S. Maria della Misericordia - Department of Medicine, University of Udine); Zalla T*, Cantafio S, Feroci F, Romoli L, Giudicissi R (SOC Chirurgia Generale, Ospedale Santo Stefano, Prato); Picciariello A*, Papagni V, Dibra R, Picciariello A, Altomare DF (Policlinic Consortium Hospital - University Of Bari); Pinotti E*, Montuori M, Baronio G (Policlinico San Pietro); Tonini V*, Sartarelli L, Gori A, Cervellera M (Policlinico Santorsola-Malpighi); Lapolla P*, Sapienza P, Brachini G, Cirillo B, Zambon M, Mingoli A (Policlinico Umberto I); Pascariello A*, Boccia L, Benedetti S, Mantovani G, De Angelis M (S.C.. Chirurgia Generale Mini Invasiva e D’Urgenza ASST Mantova Carlo Poma); Ferrara F* (Department of Surgery, San Carlo Borromeo Hospital, ASST Santi Paolo e Carlo, Milan); Testa V*, Borghi F, Maione F, Pruiti Ciarello V, Giraudo G (Santa Croce E Carle Hospital, Cuneo); Agresta F*, Cestaro G, Prando D, Cavallo F, Zese M (Santa Maria Regina Degli Angeli - Ulss 5 Polesana, Adria); Cillara N*, Sechi R, Cardia R, Cannavera A, Putzu G (Ss. Trinita Hospital); Frongia F*, Pisanu A, Delogu D, Esposito G, Podda M (University Hospital Of Cagliari). Iossa A*, De Angelis F, Boru C, Silecchia G. (Sapienza, polo pontino); Palini GM*, Garulli G, Veneroni S (Ospedale Infermi di Rimini); ​​Tammaro P*, Maida P (Unit of general Surgery, Ospedale del Mare, Napoli).

**Jamaica (JM):** Leake PA*, Wanliss MG (University Hospital Of The West Indies).

**Japan (JP):** Iwao Y*, Sato K, Chiyonobu N, Imamura H, Yamazaki S (Ohta Nishinouchi Hospital); Watanabe M (Toho University Ohashi Medical Center).

**Jordan (JO):** Qasem A*, Ayasra F, Al Dahabrh S, Khaled A (Al-Basheer Hospital); Alsaafin S*, Al-Thunaibat A, Olaywah D, Alqudah S, Alqawasmi S (Albashir Hospital); Khamees A*, Guboug A, Es Salim M, Althwabteh T, Bani Khaled H (Zarqa New Governmental Hospital); El-Hammuri N* (Hashemite University); Aljesrawi A* (University of Jordan).

**Libya (LY):** Alamaadany F*, Eljareh M, Al Gasi AEJ, Alsaeiti S, Alkhafeefi AS (Aljala Hospital); Suliaman T* (Crown Of Health Clinic); Alanasri AHA*, Haroun ABA, Haron A (Ghadames General Hospital); Kilani AI*, Ahmed M (Grand Surman Clinic); Alawami M*, Alawami A, Albashri M, Abusannuga M, Malek A (Medical Care Clinic); Jwaili N*, Aldenfria A, Jwaili N, Elzwawi F (Misurata Medical Center); Almugaddami A*, Egdeer ASA, Masoud M, Alazzabi B, Alezabi B (Nalut Central Hospital); Shuwayyah A*, Alkamkhe AAS, Aboulqasim I, Atiyah H (Sabratha Teaching Hospital); Alfagi RAA*, Abdulmula A (Shorouk Clinic); Bouhuwaish A*, Samer A, Salim R (Tobruk Medical Center); Aboazamazem H*, Almiqlash B, Biala M, Alganimi W, Ghamgh R, Ben Omar N, Alsoufi A(Tripoli Central Hospital); Aldreawi M*, Saleim N, Sowan F, Saleem H (Zliten Medical Centre), Ahmed Aqueelah* (Al Thawra Teaching Hospital).

**Lithuania (LT):** Samalavicius NE*, Aliosin O, Dailidenas S (Klaipeda University Hospital); Dulskas A*, Buckus B, Kuliesius Z, Bradunaite R (Republic Vilnius University Hospital).

**Mexico (MX):** Dominguez-Rosado I*, Buerba GA, Posadas-Trujillo OE, Alfaro-Goldaracena A, Cortes R, Mercado MA (Instituto Nacional De Ciencias Medicas Y Nutricion Salvador Zubiran); Beristain-Hernandez JL*, Mora-Munoz VS, Mena-Bedolla JM, Palacios Ramirez AR, Astorga Medina MM (La Raza National Medical Center).

**Netherlands (the) (NL):** Van Aert G*, Ombashi S, Spillenaar Bilgen R, Vos D (Amphia); Besselink M*, Alberts V, Busch O, Bemelman W, Boermeester M (Amsterdam Universitair Medische Centra); Daams F*, Gordinou De Gouberville M (Amsterdam Universitair Medische Centra Vumc Site); Van Duijvendijk P*, De Graaff M, Baaij J, Gans S, Bos K (Gelre Ziekenhuizen Apeldoorn); Goudsmit B *, Den Dekker B, Braat A (Leiden University Medical Center); Kuijpers A*, Breukers S (Onze Lieve Vrouwe Gasthius Hospital Amsterdam); Borel Rinkes I, Andel D* (University Medical Center Utrecht).

**New Zealand (NZ):** Hayes T*, Carson D, Bhat S, van der Have J, Anderson C, Bissett I, Windsor J (Auckland City Hospital); Elliott BM*, Scowcroft H, Mclauchlan J, Ritchie D, Jeffery F, Connor S (Christchurch Hospital); Xu W*, Varghese C, Mashlan H, Thirayan V, Ly J (Waikato Hospital); Mcguinness MJ*, Ferguson L, Watt I, Harmston C (Whangarei Hospital).

**Nigeria (NG):** Akinmade A*, Adeyeye A, Enoch E, Kayode-Nissi V (Afe Babalola University, Ado-Ekiti Abuad Multi-System Hospital); Ogundele I*, Ayoade BA, Adekoya A, Nwokoro C, Opadeyi A (Olabisi Onabanjo University Teaching Hospital); Adeyeye A*, Yusuf A, Ojajuni A, Adepoju O (University Of Ilorin Teaching Hospital (Uith)); Muhammad Dauda Maigatari*, Keffi Mubarak Musa, lawal Khalid, Muhammad Daniyan (Ahmadu Bello University Teaching Hospital).

**Philippines (the) (PH):** Salonga D*, Sael NA, Rey CM, Pestano M, Tan D (De Los Santos Medical Center); Bangayan NR*, Sy DK, Ang D, Bernardo E, Chua JP (The Medical City).

**Saudi Arabia (SA):** Alharthi M*, Bukhari W, Bakier Mohammed K, Al Athath S, Ghunaim M (International Medical Center); Saiedi H*, Sultan N (King Abdullah Medical Complex, Jeddah); Farsi A*, Basendowah M, Alharthi M, Ghunaim M, Malibary N (King Abdulaziz University Hospital); Jaloun H*, Altalhi Db, Organjee A, Moamena M, Al Zaidi TM (King Fahd Armed Forces Hospital); Alyami M*, Alqannas M, Al-Urfan M, Elawad A, Alawadhi A (King Khalid Hospital); Alalawi Y*, Alqarni A, Alqahtani B, Alayed A, Alsobaie K, Adi H (North West Armed Forces Hospital); Malibary N, Elhaj M, Dehlawi A, Behairy G, Khaled I (Saudi German Hospital Jeddah).

**Serbia (RS):** Kmezic S*, Radenkovic D, Aleksic L, Markovic V, Pejovic I, Antic A (Clinic For Digestive Surgery - First Surgical Clinic); KalkanM*, Vujanovic Gadjanski O, Dusan S, Marčetić B (General Hospital Pancevo).

**Singapore (SG):** Thiruchelvam N*, Chiow AKH, Lee LS, Mun DYC (Changi General Hospital); Tan EK*, Koh YX, Loh WL, Wang Z, Chan CY (Singapore General Hospital).

**South Africa (ZA):** Kloppers C*, Almgla N, Bernon M, Kahn M, Karimbocus N (Groote Schuur Hospital).

**Spain (ES):** Roldan Villavicencio JI*, Goitia V, Gutierrez Rios RD, Garcia Ruiz S, Lopez Deogracias M (Clinica Asunción); Turrado-Rodriguez V*, Morales X, Hessheimer A, Termes Serra R, Beltran De Heredia J (Hospital Clinic De Barcelona); Trujillo-Diaz J*, Herreros-Rodríguez J, Montes-Manrique M, De Andres-Asenjo B, Beltrán-Heredia J (Hospital Clinico Universitario De Valladolid); Gimenez Maurel T*, Utrilla Fornals A, Martin Anoro LF (Hospital General San Jorge); Cortese S*, Perez Diaz MD, Ballón M, Morote M, Cebolla Rojas L (Hospital General Universitario Gregorio Marañón); Oliver Guillen JR*, Lopez De Fernandez A, Del Campo Lavilla M, (Complejo Asistencial Soria); Mora-Guzmán I* (Hospital Santa Bárbara); Escartin A*, Pinillos A, Vela Polanco FF, Jara Quezada JH, Muriel Alvarez P (Hospital Universitari Arnau De Vilanova); Tur-Martinez J*, Camps J, Herrero E, Garcia-Domingo MI, Cugat Andorra E (Hospital Universitari Mutua Terrassa); Crespi Mir A*, Claramonte Bellmunt O, Vicens Arbona JC, Fernandez Burgos IR (Hospital Universitari Son Llatzer); Prieto M*, Sarriugarte Lasarte A, Marin H, Tellaeche De La Iglesia M, Ocerin Alganza O (Hospital Universitario De Cruces); Salinas Gomez J*, Ramos-Martin P, Urbieta A, Nasimi Sabbagh R, Castell Gomez JT (Hospital Universitario La Paz); Serrablo A*, Paterna -Lopez S, Gutiérrez-Díez M, Abadía-Forcen MT, Serradilla-Martín M (Hospital Universitario Miguel Servet); Duran Muñoz-Cruzado VM*, Pareja Ciuro F, Perea Del Pozo E, Aparicio Sanchez D, Dios-Barbeito S (Hospital Universitario Virgen Del Rocio); Marenco De La Cuadra B*, Retamar Gentil M, Reguera-Rosal J, Infantes Ormad M, Lopez-Ruiz JA (Hospital Universitario Virgen Macarena); Landaluce-Olavarria A*, Zevallos-Quiroz JC, Barrutia Leonardo J, Emaldi A, Begona E (Hospital Urduliz); Balciscueta Coltell I*, Sebastian M, Martinez Ramos S, Martinez Alcaide S, Lorenzo Perez J (La Ribera University Hospital); Martinez Insfran LA*, Lopez-Morales P, Gimenez Frances C (Reina Sofia University General Hospital); Rahy-Martin A*, Pelloni M, Ortiz-Lopez D, Benet-Muñoz O, Pinero-Gonzalez L (University Hospital Of Gran Canaria Dr. Negrin); Alconchel F*, Nicolas-Lopez T, Rodrigues K, Cascales Campos PA, Gomez-Bosch F, Ramirez Romero P (Virgen De La Arrixaca University Hospital (Imib-Arrixaca)).

**Sudan (the) (SD):** Ibrahim M*, Hamid HKS, Idres R (Kuwaiti Specialized Hospital); Idris M*, Mohammed O (Military Medical Hospital).

**Syrian Arab Republic (SY)**: Ayran S*, Sinan AH, Kouli O (Hisham Sinan Hospital).

**Turkey (TR)**: Ozben V*, Aytac E, Aliyeva Z, Mutlu AU (Acibadem Mehmet Ali Aydinlar University, Atakent Hospital); Bilgin IA*, Karahasanoglu T, Hamzaoglu I, Bozkirli B (Acibadem Mehmet Ali Aydinlar University, Maslak Hospital); Uprak TK*, Kotan T, Coskun M (Marmara University Research And Education Hospital); Kara Y*, Somuncu E, Kocatas A, Bozkurt MA (Tr, Health Sciences University, Kanuni Sultan Suleyman Training And Research Hospital); Demirli Atici S*, Kaya T, Sert I, Emiroglu M (University Of Health Sciences Tepecik Training And Research Hospital).

**United Arab Emirates (the) (AE)**: Jaffar M*, Younis MU, Aziz T, Ikram F (Mediclinic City Hospital); Sandal M*, Al Madhloum Al Suwaidi F, Alshaikh MO, Saber A, Khammas A (Rashid Hospital).

**United Kingdom (UK):** Nessa A*, Jardine R, Nicol L, Clark C, Mcgee A, Alkari B (Aberdeen Royal Infirmary); Feretis M*, Antakia R, Georgiades F, Moneim J, O’Neill R, Balakrishnan A (Addenbrookes Hospital); Lunevicius R*, Sud A, Moutsos I, Gomez D, Shahid S (Aintree University Hospital); Majeed T*, Ibrahim WKG, Kadum K, Melia R, Magee C (Arrowe Park Hospital); Chicken DW*, Kumar S, Alshibshoubi M (Basildon University Hospital); van Laarhoven S*, Dewi F, Williams J, Byrne B, Wilkerson P (Bristol Royal Infirmary); Tang CB*, Farhangmehr N, Jonas A, Charavanamuttu V, Almeida K (Broomfield Hospital); Efthimiou E*, Boardley J, White A, Butt MA (Chelsea & Westminster Hospital Nhs Foundation Trust); Menzies D*, Gundkalli Z, Hassanzadeh-Baboli D (Colchester Hospital); Jones O*, Mistry P, Saha S, Gerrard A, Evans J (Countess Of Chester Hospital); Rajeev S*, Ali W, Ross E, Gilliam A (Darlington Memorial Hospital); Hitchins C*, Emslie K, Spellar K, Sked H, Briggs C (Derriford Hospital); Brown L*, A Hemadasa N, Apollos JR (Dumfries & Galloway Royal Infirmary); Belgaumkar A*, Tawfik A, Brewin L, Oyewole B (East Surrey Hospital); Wadhawan H*, Massie E, Rutherford D, Mcgivern K, Mcelroy L (Forth Valley Royal Hospital); De’Ath HD*, Tobbal M, Nagendram S (Frimley Park Hospital); Patel P*, Handa S, Houghton G, Sundaralingam SS, Parker J (Furness General Hospital); Morgan R*, Gala T, Ibrahim S, Harby R, Abdelkarim M (Glan Clwyd Hospital); Holroyd D*, Carson D, Thomas R, Mclennan E, Boardley R, Jamieson NB (Glasgow Royal Infirmary); Ebied H*, Gossage J, Davies A, Wheatstone S (Guys And St Thomas Hospital); Jawad Z*, Jiao L, Rajagopal P, Sodergren M (Hammersmith Hospital, Imperial College); Lami M*, Gacaferi H, Wiberg A, Bond-Smith G (John Radcliffe Hospital); Gemmill E*, Lenzi E, Sapre D, Herrod P, Boyd-Carson H (Kings Mill Hospital); Garcea G*, Issa E, Jackson A, Fashina T, Pan H (Leicester General Hospital); Farquharson B*, Shafiq H, Emanuel O, Mahdi S, Jeyarajah S (Lister Hospital); Finch L*, Whiting G, Pigott L, Martin J, Siriwardena AK (Manchester Royal Infirmary); Beatson K*, Abawi L, Lam W, Rea W, Andrews B (Medway Maritime Hospital); Al-Sarireh B*, Soliman F, Burridge J, Jenvey C, Hammoda M (Morriston Hospital); Hollyman M*, Merker L, Richards J, Sukumaran V, Rogers S (Musgrove Park Hospital); Payne C*, Bibi S, Raza K, Ul Ain N (Ninewells Hospital); Dronamraju S, Patil S, Nachimuthu S, Ravindran S, Patel S (Pinderfields Hospital); Ivanov B*, Patel M, Ejtehadi F, Jebamani J (Princess Alexandra Hospital); Akhter Rahman MM*, Woodun H, De Prendergast A, Afzal A, Bota E (Princess Of Wales Hospital); Gupta A*, Abdul SR, Karmarkar R, Crockett E, Evans L, Appleton B (Princess Of Wales Hospital, Bridgend); Griffiths E*, Dada O, Kulkarni R (Queen Elizabeth Hospital Birmingham); Albirnawi H*, Gravestock P, Vincenti C, Taribagil S, Dent B (Queen Elizabeth Hospital (Gateshead)); Tse C*, Clayton B, Burdekin E, Bannister L, Alam I (Royal Albert Edward Infirmary); Gray J*, Mactier M, Pollock A, Gough V (Royal Alexandra Hospital); Kanchustambam SR*, Ridgway M, Arujunan K (Royal Blackburn Teaching Hospital); Gopalswamy S*, Monteiro De Barros J, Lyons T, Griffith D (Royal Cornwall Hospital); Awan AK*, Latif J, Bandlamudi N, Bhatti I (Royal Derby Hospital); Raptis DA*, Machairas N, Pissanou T, Mestre-Costa J, Hidalgo Salinas C, Pollok JM (Royal Free Hospital); Al-Ardah M*, White A, Watson-Jones E, Rontree-Carey T, Boyce T (Royal Gwent Hospital); Hawkin P*, Elmaradny A, Ross K, Adu-Peprah E, Pinto K (Royal Lancaster Infirmary); Dunne D*, Mccready R, Nita G, Szatmary P, Tay VL, Rajput K (Royal Liverpool And Broadgreen Hospital); Rajendran I*, Chaudhury M, Zambas G (Royal Preston Hospital); Swaminathan C*, Atif QAA, Barrow T, Williams O, Malik A (Royal Sussex County Hospital); Conroy S*, Lindley S, Gilmore K, Boden E, Richards SK (Royal United Hospital); Hraishawi I*, Polak P, Mclaughlin D, Deeny D, Shuttleworth R, Harris A (Royal Victoria Hospital, Belfast); Peilober-Richardson A*, Morris GC, Sara X, Almourad H, Ang Y (Salford Royal Foundation Trust); Smyth R*, Ding D, Foster J, Bond A (Salisbury District Hospital); Kumar Y*, Ahmad A, Radoi D, Alkaili-Alyamani A, Balakrishnan S (Sandwell General Hospital); Satchidanand RY, Danwaththa Liyanage AS*, Blake I, Ransome M, Weerasinghe C (Southport And Formby District General Hospital); Kenington C*, Mayo K, Mohammed M (St Georges Hospital); Cockbain AJ*, Peckham-Cooper A, Mccauley G, Gordon C, Smith A (St James's University Hospital); Hawkins W*, Chakravartty S, Baillie C, Kenny R (St Richards Hospital); Kumar A*, Koimtzis G, Bellamy E, Menon A (Stepping Hill Hospital, Stockport Nhs Foundation Trust); Kanakala A*, Nevins EJ, Madhavan A, Thulasiraman S, France K (The James Cook University Hospital); O’Connor A*, Idama D, Raslan C, Sridhar S, Parveen M (The Royal Oldham Hospital); Mubashar T*, Jarvis S, Cakmak I, Wright C, Andrews S (Torbay Hospital); Abdelsaid K* Abdul Aal Y, Jayasankar B, Morilla J, Shehata M, Subba N (Tunbridge Wells Hospital); Tewari N*, El-Sayed C, Somaie D, Beheiry N, Douka E (University Hospital Coventry); Arumugam S*, Wijetunga I, Leivers E, Ibrahim B, Khan K (University Hospital Of North Durham); Wheat J*, Christopher J, Barnett R (University Hospital Of Wales); Elberm H*, Booker J, Ashai S, Berry D (University Hospital Southampton); Luhmann A*, Sgro A, Rashid MM, Galea M, Jeyakumar J (Victoria Hospital Kirkcaldy); Marriott P*, Zafar S, Baker A, Yershov D, Galanopoulos G (Warwick Hospital); Gupta A*, Jordan R, Peinado Garcia C, Anyaugo N (Weston General Hospital); Bath MF*, Evans J, Omatseye J, Roberts L, Argyriou EO, Machesney M (Whipps Cross Hospital); Parmar C*, Clark S, Khalil H, Unsworth S (Whittington Health); Mlotshwa M*, Ayoub N, Aboelkhair A, Iosif E, Mohamed N (Worthing Hospital); Reynolds E*, Mackender E, Robinson D, Mufti W (Wythenshawe Hospital).

**United States of America (the) (US):** Fischkoff K*, Coleman N (Columbia University Irving Medical Centre); Anantha Sathyanarayana S*, Deutsch G, Giangola M, Lin D, Weiss M (Donald And Barbara Zucker School Of Medicine At Hofstra/Northwell); Chung C*, Nguyen A, Mueller J, Dabit M, Gordon J, McGuire E (HCA Swedish Medical Center); Rashid O*, Georgi E, Gallo M (Holy Cross Hospital); Kunstman JW*, Peters NV, O’Connor R, Bhattacharya B (Yale School of Medicine); Onkendi E*, Santos AP, Richmond R, Warren M, Zhang K (Texas Tech University Health Sciences Center); Broderick R*, Clary B, Horgan S, Doucet J, Liepert A (UC San Diego Health); Harmon L*, McCall C (University Of Colorado Hospital); Sham JG*, Williams E, Labadie KP, Clark NM, Dickerson LK (University Of Washington Medical Centre); Hammill CW*, Williams G, Kushner B, Cos H, Zarate Rodriguez J (Washington University In St. Louis); Bailey K* (West Virginia University Medical Corporation).

**Yemen (YE):** Al-Raimi IMN*, Al-Zazay K, Ahmed Mohammed Al-Mahdi S, Mohammed Aldowbli S (Al-Khamseen Hospital); Al-Shehari M*, Shream S, Al-Ameri S, Aeed M, Al-Naggar H (Al-Thawra Modern General Hospital, Sana’a); Aldawbali M*, Alsayadi R, Alsayadi M (Royal Hospital).
