# Corrigendum to: Global overview of the management of acute cholecystitis during the COVID-19 pandemic (CHOLECOVID study)

**DOI:** 10.1093/bjsopen/zrac088

**Published:** 2022-06-23

**Authors:** CHOLECOVID Collaborative

*BJS Open*, https://doi.org/10.1093/bjsopen/zrac052

In the originally published version of this manuscript, there were authorship errors. The following changes have been made to the article:

- The author byline has been corrected to only include the group author.
- The group author affiliations have been corrected – a second affiliation has been added.
- Spelling errors in three contributors’ names have been corrected.
- The South Africa and Spain collaborators have been added to the Collaborators section of the main manuscript (previously omitted in error).
- The supplementary file has been resupplied with the same collaborator group corrections.

