# Supplementary material for: Global overview of the management of acute cholecystitis during the COVID-19 pandemic (CHOLECOVID study)
Source: BJS Open. 2022 May 4;6(3):zrac052. doi: 10.1093/bjsopen/zrac052 (PMC9071082; doi:10.1093/bjsopen/zrac052)
Supplement: zrac052_Supplementary_Data [file zrac052_supplementary_data.zip › Supplementary_material_UPDATED_confirmed.docx]

**Appendix: CHOLECOVID Collaborative Authorship**

## CHOLECOVID Collaborative

**Writing group**: Harry V M Spiers^*^, Omar Kouli^*^, Waheed U Ahmed, Rebecca Varley, Daniel Ahari, Leah Argus, Kenneth A McLean, Sivesh K Kamarajah, Peter Coe, Ewen A Griffiths, Anthony KC Chan, Christian Macutkiewicz, Saurabh Jamdar, Michael Wilson, Catherine Fullwood, Giles Toogood, Ajith K Siriwardena (senior author & overall guarantor).

**Joint first authors who contributed equally*

**Statistical analysis**: Omar Kouli, Kenneth McLean, Catherine Fullwood.

**Operations committee**: Daniel Ahari, Leah Argus, Rebecca Varley, Harry V M Spiers, Omar Kouli, Waheed Ahmed, Andrew Gilchrist, Matthew Goldsworthy, Majid Rashid.

**National Leads:** P Pockney (Australia), Varela J (El Salvador), Brindl N (Germany), Ramirez J (Guatemala), Marafante C (Italy), Iwao Y(Japan), Ghzawi A (Jordan), Elhadi M (Libya), Gacaferi H (The Netherlands), Varghese C (New Zealand), Adeyeye A(Nigeria),Alser O (Palestine), Teh C (Philippines), Prieto M (Spain), Hasan A (United Arab Emirates), Al-Naggar H (Yemen).

**Collaborators**

*denotes principal investigator at each site.

**Argentina (AR):** Salgado R*, Veracierto F, Lancelotti T, Solinas D, Oddi R (Cemic (Centro De Educacion Medica E Investigaciones Clinicas)); Garcia FW*, Mazza Diez E, Andrade Ramirez MR, Bracco R, Fernandez D (Clinica Pueyrredon); Maraschio MA*, Obeide L, Giordano E, Alcaraz A, Marani MA (Hospital Privado Universitario De Cordoba); Aguirre N*, Luna F, Francesconi M, Chiham F, Ramos Cossio R (Petrona V. De Cordero); Alvarez FA*, Pantoja Pachajoa DA, Mandojana F (Clinica Universitaria Reina Fabiola); Merlo IG*, Gonzalez MH, Cervelo G, Puma R, Vardaro GF (Sanatorio San Justo).

**Australia (AU):** Davis A*, Jurat D, Guenoff C, Raubenheimer K,​​ Goddard K (Armadale Health Service); Brown K, Wegrecki KJ, Cheung HYC*, Yang M (Canterbury Hospital); Cheung H*, Siddiqui J, Ahn JH, Huynh R (Concord Repatriation General Hospital); Lam YH*, Afzal M, Ong BS, Chua MYM, Ly K, Thomson JE, Watson D (Flinders Medical Centre); Dawson AC*, Drane A, Van Ruyven S, Lun EWY (Gosford Hospital); Pockney P*, Ferguson M, Jeong JY, De Silva C, Wills V (John Hunter Hospital); Gundara J*, Mccourt E, Bong C, Tabone R, Wong WJ (Metro South Health (Redland/Logan Hospitals)); Gray A*, Koh D, Pollock M, Singhal S, Smith R (Monash Medical Centre); Dudi-Venkata NN*, Kanhere H, Stranz C, Seow W, Mansour LT, Wormald J (Royal Adelaide Hospital); Loveday BPT*, Thomson B, O’Donnell T, Milenkovski N (Royal Melbourne Hospital); Herath M*, Trochsler M, Farfus A, Maddern G, Bunjo Z, Kuan LL, Atanasov G (The Queen Elizabeth Hospital); Dawson A*, Drane A, van Ruyven S, Lun E (Wyong Public Hospital).

**Azerbaijan (AZ):** Samadov E*, Namazov I, Asgarov M, Ibrahimli A (Leyla Medical Center).

**Bahrain (BH):** Srinivasan M*, Saeed MF, Aljawder H, Juma I (King Hamad University Hospital).

**Brazil (BR):** Coimbra FJ*, Marques N (A.C. Camargo Cancer Center); Casteleins WA*, Petruzziello A, Jabur G, Rodriguez JFP, Buso PL (Hospital Universitario Cajuru).

**Canada (CA):** MacKenzie S*, Hsiao M, Sljivic I, Tecson A (Royal Columbian Hospital); Karanicolas PJ*, Roke R, Moon J, Butler EV (Sunnybrook Health Sciences Centre).

**Chile (CL):** Riquelme F*, Yanez M, Catan F, Uribe M (Hospital Del Salvador); Carriel F*, Oppliger F, Paredes A, Daroch D, Aguayo JC (Hospital Padre Hurtado).

**Colombia (CO):** Perez Rivera CJ*, Acosta Buitrago LM, Kadamani Abiyomaa A, Mosquera Paz MS, Cabrera P, Corso J (Fundacion Cardioinfantil - Instituto De Cardiologia).

**Cyprus (CY):** Ozcay N*, Ozant A, Arslan K, Besim H, Almezghwi H (Near East University Hospital).

**Egypt (EG):** Azzam AY* (Al-Azhar University Hospitals); Bessa S, El-Sayes I, Badawy A, Wael M (Alexandria University Medical Centre); El-Gendi A*, Azab MA (Cairo University Hospitals); Fayed M (El-Mahalla Hepatology Teaching Hospital); El Kassas M*, Gamal M, Tawheed A, Al Shafie A (Endemic Medicine Department,Helwan University); Emile S*, Elfallal A, Elfeki H, Shalaby M, Sakr A (Mansoura University Hospital); Elbahnasawy M*, Shama M, Abdel-Elsalam W, Abd-ElsalamS (Tanta University Hospital).

**El Salvador (SV):** Escobar Dominguez JE*, Medrano F, Gaitan S, Escalon Gonzalez OM (Central Military Hospital); Alfaro Varela JC*, Cea M, Interiano M (Instituto Salvadoreno Del Seguro Social (Salvadoran Institute Of Social Security)); Cabrera B* (Hospital Nacional San Juan de Dios).

**France (FR):** Lakkis Z*, Georges P, Antonot C, Magnin J (University Hospital Of Besançon).

**Germany (DE)**: Kamphues C*, Lauscher JC, Schineis C, Loch FN, Lee LD, Beyer K (Charite University Medicine, Campus Benjamin Franklin).

**Greece (GR):** Bouchagier K*, Galanis I, Bartziotas D (General Hospital Of Athens Evaggelismos); Lostoridis E*, Tourountzi P, Nagorni EA (Kavala General Hospital); Charalabopoulos A*, Baili E, Kyros E, Vagios I, Skotsimara A, Liakakos T, Alexandrou A, Papalampros A (Laiko General Hospital); Papadopolous V*, Tooulias A, Kentarchos I, Christou C, Tsoulfas G (Papageorgiou General Hospital).

**Guatemala (GT):** Tale-Rosales LF*, Lopez Muralles I, Melendez H, Bran G, Monroy Mahecha FA (General Hospital Dr. Juan Jose Arevalo Bermejo, Guatemalan Institute Of Social Security); Contreras JR*, Porras DE, Paiz E, Soto ER, Ixcayau Hernandez JR (Instituto Guatemalteco De Seguridad Social).

**India (IN):** Gupta A*, Rajput D, Kumar N, Mani R*, Kant R (All India Institute Of Medical Sciences Rishikesh); Sonkar AA*, Anand A, Agrawal MK, Gaurav K, Tripathi M (King Georges Medical University); Sikora S*, Bharathy K, Kumar Rangapa M, Khuller DS, K SK (Sakra World Hospital); Bhojwwani R*, Ayyar S, Jain N (Santokba Durlabhji Memorial Hospital); Mehraj A*, Hussain F, Nazir I, Shah M, Chowdri NA (Sher I Kashmir Institute Of Medical Sciences Srinagar).

**Iraq (IQ):** Hilmi A* (Ibn Sina Hospital).

**Italy (IT):** Argenio G*, Atelli P, Palladino E, Armellino MF (Aou San Giovanni Di Dio E Ruggi Daragona); Tamini N*, Nespoli LC, Degrate L, Angrisani M, Carissimi F, Bordoni P (ASST Monza - Ospedale San Gerardo); Fleres F*, Bordoni P, Clarizia G, Spolini A, Franzini M (General Surgery Unit,Ospedale Civile di Sondrio,  ASST Valtellina E Alto Lario, Sondrio, Italy); Cucinotta E*, Badessi G, Mazzeo C, Viscosi F, Pintabona G (General and Emergency Surgery Unit, Azienda Ospedaliera Policlinico Universitario G. Martino, University of Messina, Messina, Italy); Campagnaro T*, Poletto E, Turri G, Ruzzenente A, Conci S, Guglielmi A (Azienda Ospedaliera Universitaria Integrata Verona); Feo C*, Fabbri N, Fazzin M, Giaccari S, Feo CV (Azienda Usl Di Ferrara); Massani M, Pelizzo P, Colella M, Tutino R* (Chirurgia 1 - Azienda ULSS2 Marca Trevigiana - Ospedale Regionale Treviso); Cappellacci F*, Medas F, Canu GL, Erdas E, Calò PG (Chirurgia Generale Polispecialistica Aou); Porcu A*, Perra T, Scanu AM, Feo CF, Fancellu A (Clinica Chirurgica Azienda Ospedaliero Universitaria (Aou) - Sassari); Germani P*, Giunta C, Biloslavo A, Abdallah H, Aizza G (Clinica Chirurgica Cattinara University Hospital); Barberis A*, Belli F, Santoliquido M, Filauro M (E.o. Ospedali Galliera, Genoa); Canonico G*, Nelli T, Di Martino C, Capezzuoli L, Anastasi A, Bressan L, Cortinovis S, Nagliati C (Hospital San Giovanni Di Dio); Colombo F*, Ferrario L, Bondurri A, Guerci C, Maffioli A (L. Sacco University Hospital); Catena F*, Perrone G, Giuffrida M, Morini A, Annicchiarico A (Maggiore Hospital); Gallo G*, Carpino A, Ferrari F, De Paola G, Sammarco G (Operative Unit Of Digestive Surgery, University “Magna Graecia” of Catanzaro, Catanzaro, Italy); Callari C*, Licari L, Sorce V, Di Miceli D (Ospedale Buccheri La Ferla Hospital); Lovisetto F*, Zonta S, Lovisetto F (Ospedale Castelli Verbania); Chessa A*, Fiorini A (Ospedale Civile San Giovanni Di Dio); De Manzoni Garberini A*, Angelini E (Ospedale Civile Spirito Santo); Marafante C*, Moggia E, Murgese A, Mungo S, Birolo SL, Garino M (Ospedale degli Infermi, Rivoli); Pipitone Federico NS*, Muratore A, Lunghi EG, Calabro M (Ospedale Edoardo Agnelli); Cianci P*, Enrico R, Capuzzolo S, Cafagna L, Minafra M (Ospedale Lorenzo Bonomo Chirurgia); Sasia D*, Gattolin A, Migliore M, Rimonda R, Travaglio E (Regina Montis Regalis Hospital, Mondovì); De Marco G*, Elter C, Bargellini T, D’amico S, Zambonin D, Caponi A (Ospedale San Giuseppe Di Empoli Chirurgia Generale); Calini G*, Puggioni A, Bresadola V (University Hospital S. Maria della Misericordia - Department of Medicine, University of Udine); Zalla T*, Cantafio S, Feroci F, Romoli L, Giudicissi R (SOC Chirurgia Generale, Ospedale Santo Stefano, Prato); Picciariello A*, Papagni V, Dibra R, Picciariello A, Altomare DF (Policlinic Consortium Hospital - University Of Bari); Pinotti E*, Montuori M, Baronio G (Policlinico San Pietro); Tonini V*, Sartarelli L, Gori A, Cervellera M (Policlinico Santorsola-Malpighi); Lapolla P*, Sapienza P, Brachini G, Cirillo B, Zambon M, Mingoli A (Policlinico Umberto I); Pascariello A*, Boccia L, Benedetti S, Mantovani G, De Angelis M (S.C.. Chirurgia Generale Mini Invasiva e D’Urgenza ASST Mantova Carlo Poma); Ferrara F* (Department of Surgery, San Carlo Borromeo Hospital, ASST Santi Paolo e Carlo, Milan); Testa V*, Borghi F, Maione F, Pruiti Ciarello V, Giraudo G (Santa Croce E Carle Hospital, Cuneo); Agresta F*, Cestaro G, Prando D, Cavallo F, Zese M (Santa Maria Regina Degli Angeli - Ulss 5 Polesana, Adria ); Cillara N*, Sechi R, Cardia R, Cannavera A, Putzu G (Ss. Trinita Hospital); Frongia F*, Pisanu A, Delogu D, Esposito G, Podda M (University Hospital Of Cagliari). Iossa A*, De Angelis F, Boru C, Silecchia G. (Sapienza, polo pontino); Palini GM*, Garulli G, Veneroni S (Ospedale Infermi di Rimini); ​​Tammaro P*, Maida P (Unit of general Surgery, Ospedale del Mare, Napoli).

**Jamaica (JM):** Leake PA*, Wanliss MG (University Hospital Of The West Indies).

**Japan (JP):** Iwao Y*, Sato K, Chiyonobu N, Imamura H, Yamazaki S (Ohta Nishinouchi Hospital); Watanabe M (Toho University Ohashi Medical Center).

**Jordan (JO):** Qasem A*, Ayasra F, Al Dahabrh S, Khaled A (Al-Basheer Hospital); Alsaafin S*, Al-Thunaibat A, Olaywah D, Alqudah S, Alqawasmi S (Albashir Hospital); Khamees A*, Guboug A, Es Salim M, Althwabteh T, Bani Khaled H (Zarqa New Governmental Hospital); El-Hammuri N* (Hashemite University); Aljesrawi A* (University of Jordan).

**Libya (LY):** Alamaadany F*, Eljareh M, Al Gasi AEJ, Alsaeiti S, Alkhafeefi AS (Aljala Hospital); Suliaman T* (Crown Of Health Clinic); Alanasri AHA*, Haroun ABA, Haron A (Ghadames General Hospital); Kilani AI*, Ahmed M (Grand Surman Clinic); Alawami M*, Alawami A, Albashri M, Abusannuga M, Malek A (Medical Care Clinic); Jwaili N*, Aldenfria A, Jwaili N, Elzwawi F (Misurata Medical Center); Almugaddami A*, Egdeer ASA, Masoud M, Alazzabi B, Alezabi B (Nalut Central Hospital); Shuwayyah A*, Alkamkhe AAS, Aboulqasim I, Atiyah H (Sabratha Teaching Hospital); Alfagi RAA*, Abdulmula A (Shorouk Clinic); Bouhuwaish A*, Samer A, Salim R (Tobruk Medical Center);  Aboazamazem H*, Almiqlash B, Biala M, Alganimi W, Ghamgh R, Ben Omar N, Alsoufi A(Tripoli Central Hospital); Aldreawi M*, Saleim N, Sowan F, Saleem H (Zliten Medical Centre), Ahmed Aqueelah* (Al Thawra Teaching Hospital).

**Lithuania (LT):** Samalavicius NE*, Aliosin O, Dailidenas S (Klaipeda University Hospital); Dulskas A*, Buckus B, Kuliesius Z, Bradunaite R (Republic Vilnius University Hospital).

**Mexico (MX):** Dominguez-Rosado I*, Buerba GA, Posadas-Trujillo OE, Alfaro-Goldaracena A, Cortes R, Mercado MA (Instituto Nacional De Ciencias Medicas Y Nutricion Salvador Zubiran); Beristain-Hernandez JL*, Mora-Munoz VS, Mena-Bedolla JM, Palacios Ramirez AR, Astorga Medina MM (La Raza National Medical Center).

**Netherlands (the) (NL):** Van Aert G*, Ombashi S, Spillenaar Bilgen R, Vos D (Amphia); Besselink M*, Alberts V, Busch O, Bemelman W, Boermeester M (Amsterdam Universitair Medische Centra); Daams F*, Gordinou De Gouberville M (Amsterdam Universitair Medische Centra Vumc Site); Van Duijvendijk P*, De Graaff M, Baaij J, Gans S, Bos K (Gelre Ziekenhuizen Apeldoorn); Goudsmit B *, Den Dekker B, Braat A (Leiden University Medical Center); Kuijpers A*, Breukers S (Onze Lieve Vrouwe Gasthius Hospital Amsterdam); Borel Rinkes I, Andel D* (University Medical Center Utrecht).

**New Zealand (NZ):** Hayes T*, Carson D, Bhat S, van der Have J, Anderson C, Bissett I, Windsor J (Auckland City Hospital); Elliott BM*, Scowcroft H, Mclauchlan J, Ritchie D, Jeffery F, Connor S (Christchurch Hospital); Xu W*, Varghese C, Mashlan H, Thirayan V, Ly J (Waikato Hospital); Mcguinness MJ*, Ferguson L, Watt I, Harmston C (Whangarei Hospital).

**Nigeria (NG):** Akinmade A*, Adeyeye A, Enoch E, Kayode-Nissi V (Afe Babalola University, Ado-Ekiti Abuad Multi-System Hospital); Ogundele I*, Ayoade BA, Adekoya A, Nwokoro C, Opadeyi A (Olabisi Onabanjo University Teaching Hospital); Adeyeye A*, Yusuf A, Ojajuni A, Adepoju O (University Of Ilorin Teaching Hospital (Uith)); Muhammad Dauda Maigatari*, Keffi Mubarak Musa, lawal Khalid, Muhammad Daniyan (Ahmadu Bello University Teaching Hospital).

**Philippines (the) (PH):** Salonga D*, Sael NA, Rey CM, Pestano M, Tan D (De Los Santos Medical Center); Bangayan NR*, Sy DK, Ang D, Bernardo E, Chua JP (The Medical City).

**Saudi Arabia (SA):** Alharthi M*, Bukhari W, Bakier Mohammed K, Al Athath S, Ghunaim M (International Medical Center); Saiedi H*, Sultan N (King Abdullah Medical Complex, Jeddah); Farsi A*, Basendowah M, Alharthi M, Ghunaim M, Malibary N (King Abdulaziz University Hospital); Jaloun H*, Altalhi Db, Organjee A, Moamena M, Al Zaidi TM (King Fahd Armed Forces Hospital); Alyami M*, Alqannas M, Al-Urfan M, Elawad A, Alawadhi A (King Khalid Hospital); Alalawi  Y*, Alqarni A, Alqahtani B, Alayed A, Alsobaie K, Adi H (North West Armed Forces Hospital); Malibary N, Elhaj M, Dehlawi A, Behairy G, Khaled I (Saudi German Hospital Jeddah).

**Serbia (RS):** Kmezic S*, Radenkovic D, Aleksic L, Markovic V, Pejovic I, Antic A (Clinic For Digestive Surgery - First Surgical Clinic); KalkanM*, Vujanovic Gadjanski O, Dusan S, Marčetić B (General Hospital Pancevo).

**Singapore (SG):** Thiruchelvam N*, Chiow AKH, Lee LS, Mun DYC (Changi General Hospital); Tan EK*, Koh YX, Loh WL, Wang Z, Chan CY (Singapore General Hospital).

**South Africa (ZA):** Kloppers C*, Almgla N, Bernon M, Kahn M, Karimbocus N (Groote Schuur Hospital).

**Spain (ES):** Roldan Villavicencio JI*, Goitia V, Gutierrez Rios RD, Garcia Ruiz S, Lopez Deogracias M (Clinica Asunción); Turrado-Rodriguez V*, Morales X, Hessheimer A, Termes Serra R, Beltran De Heredia J (Hospital Clinic De Barcelona); Trujillo-Diaz J*, Herreros-Rodríguez J, Montes-Manrique M, De Andres-Asenjo B, Beltrán-Heredia J (Hospital Clinico Universitario De Valladolid); Gimenez Maurel T*, Utrilla Fornals A, Martin Anoro LF (Hospital General San Jorge); Cortese S*, Perez Diaz MD, Ballón M, Morote M, Cebolla Rojas L (Hospital General Universitario Gregorio Marañón); Oliver Guillen JR*, Lopez De Fernandez A, Del Campo Lavilla M, (Complejo Asistencial Soria); Mora-Guzmán I* (Hospital Santa Bárbara); Escartin A*, Pinillos A, Vela Polanco FF, Jara Quezada JH, Muriel Alvarez P (Hospital Universitari Arnau De Vilanova); Tur-Martinez J*, Camps J, Herrero E, Garcia-Domingo MI, Cugat Andorra E (Hospital Universitari Mutua Terrassa); Crespi Mir A*, Claramonte Bellmunt O, Vicens Arbona JC, Fernandez Burgos IR (Hospital Universitari Son Llatzer); Prieto M*, Sarriugarte Lasarte A, Marin H, Tellaeche De La Iglesia M, Ocerin Alganza O (Hospital Universitario De Cruces); Salinas Gomez J*, Ramos-Martin P, Urbieta A, Nasimi Sabbagh R, Castell Gomez JT (Hospital Universitario La Paz); Serrablo A*, Paterna -Lopez S, Gutiérrez-Díez M, Abadía-Forcen MT, Serradilla-Martín M (Hospital Universitario Miguel Servet); Duran Muñoz-Cruzado VM*, Pareja Ciuro F, Perea Del Pozo E, Aparicio Sanchez D, Dios-Barbeito S (Hospital Universitario Virgen Del Rocio); Marenco De La Cuadra B*, Retamar Gentil M, Reguera-Rosal J, Infantes Ormad M, Lopez-Ruiz JA (Hospital Universitario Virgen Macarena); Landaluce-Olavarria A*, Zevallos-Quiroz JC, Barrutia Leonardo J, Emaldi A, Begona E (Hospital Urduliz); Balciscueta Coltell I*, Sebastian M, Martinez Ramos S, Martinez Alcaide S, Lorenzo Perez J (La Ribera University Hospital); Martinez Insfran LA*, Lopez-Morales P, Gimenez Frances C (Reina Sofia University General Hospital); Rahy-Martin A*, Pelloni M, Ortiz-Lopez D, Benet-Muñoz O, Pinero-Gonzalez L (University Hospital Of Gran Canaria Dr. Negrin); Alconchel F*, Nicolas-Lopez T, Rodrigues K, Cascales Campos PA, Gomez-Bosch F, Ramirez Romero P (Virgen De La Arrixaca University Hospital (Imib-Arrixaca)).

**Sudan (the) (SD):** Ibrahim M*, Hamid HKS, Idres R (Kuwaiti Specialized Hospital); Idris M*, Mohammed O (Military Medical Hospital).

**Syrian Arab Republic (SY)**: Ayran S*, Sinan AH, Kouli O (Hisham Sinan Hospital).

**Turkey (TR)**: Ozben V*, Aytac E, Aliyeva Z, Mutlu AU (Acibadem Mehmet Ali Aydinlar University, Atakent Hospital); Bilgin IA*, Karahasanoglu T, Hamzaoglu I, Bozkirli B (Acibadem Mehmet Ali Aydinlar University, Maslak Hospital); Uprak TK*, Kotan T, Coskun M (Marmara University Research And Education Hospital); Kara Y*, Somuncu E, Kocatas A, Bozkurt MA (Tr, Health Sciences University, Kanuni Sultan Suleyman Training And Research Hospital); Demirli Atici S*, Kaya T, Sert I, Emiroglu M (University Of Health Sciences Tepecik Training And Research Hospital).

**United Arab Emirates (the) (AE)**: Jaffar M*, Younis MU, Aziz T, Ikram F (Mediclinic City Hospital); Sandal M*, Al Madhloum Al Suwaidi F, Alshaikh MO, Saber A, Khammas A (Rashid Hospital).

**United Kingdom (UK):** Nessa A*, Jardine R, Nicol L, Clark C, Mcgee A, Alkari B (Aberdeen Royal Infirmary); Feretis M*, Antakia R, Georgiades F, Moneim J, O’Neill R, Balakrishnan A (Addenbrookes Hospital); Lunevicius R*, Sud A, Moutsos I, Gomez D, Shahid S (Aintree University Hospital); Majeed T*, Ibrahim WKG, Kadum K, Melia R, Magee C (Arrowe Park Hospital); Chicken DW*, Kumar S, Alshibshoubi M (Basildon University Hospital); van Laarhoven S*, Dewi F, Williams J, Byrne B, Wilkerson P (Bristol Royal Infirmary); Tang CB*, Farhangmehr N, Jonas A, Charavanamuttu V, Almeida K (Broomfield Hospital); Efthimiou E*, Boardley J, White A, Butt MA (Chelsea & Westminster Hospital Nhs Foundation Trust); Menzies D*, Gundkalli Z, Hassanzadeh-Baboli D (Colchester Hospital); Jones O*, Mistry P, Saha S, Gerrard A, Evans J (Countess Of Chester Hospital); Rajeev S*, Ali W, Ross E, Gilliam A (Darlington Memorial Hospital); Hitchins C*, Emslie K, Spellar K, Sked H, Briggs C (Derriford Hospital); Brown L*, A Hemadasa N, Apollos JR (Dumfries & Galloway Royal Infirmary); Belgaumkar A*, Tawfik A, Brewin L, Oyewole B (East Surrey Hospital); Wadhawan H*, Massie E, Rutherford D, Mcgivern K, Mcelroy L (Forth Valley Royal Hospital); De’Ath HD*, Tobbal M, Nagendram S (Frimley Park Hospital); Patel P*, Handa S, Houghton G, Sundaralingam SS, Parker J (Furness General Hospital); Morgan R*, Gala T, Ibrahim S, Harby R, Abdelkarim M (Glan Clwyd Hospital); Holroyd D*, Carson D, Thomas R, Mclennan E, Boardley R, Jamieson NB (Glasgow Royal Infirmary); Ebied H*, Gossage J, Davies A, Wheatstone S (Guys And St Thomas Hospital); Jawad Z*, Jiao L, Rajagopal P, Sodergren M (Hammersmith Hospital, Imperial College); Lami M*, Gacaferi H, Wiberg A, Bond-Smith G (John Radcliffe Hospital); Gemmill E*, Lenzi E, Sapre D, Herrod P, Boyd-Carson H (Kings Mill Hospital); Garcea G*, Issa E, Jackson A, Fashina T, Pan H (Leicester General Hospital); Farquharson B*, Shafiq H, Emanuel O, Mahdi S, Jeyarajah S (Lister Hospital); Finch L*, Whiting G, Pigott L, Martin J, Siriwardena AK (Manchester Royal Infirmary); Beatson K*, Abawi L, Lam W, Rea W, Andrews B (Medway Maritime Hospital); Al-Sarireh B*, Soliman F, Burridge J, Jenvey C, Hammoda M (Morriston Hospital); Hollyman M*, Merker L, Richards J, Sukumaran V, Rogers S (Musgrove Park Hospital); Payne C*, Bibi S, Raza K, Ul Ain N (Ninewells Hospital); Dronamraju S, Patil S, Nachimuthu S, Ravindran S, Patel S (Pinderfields Hospital); Ivanov B*, Patel M, Ejtehadi F, Jebamani J (Princess Alexandra Hospital); Akhter Rahman MM*, Woodun H, De Prendergast A, Afzal A, Bota E (Princess Of Wales Hospital); Gupta A*, Abdul SR, Karmarkar R, Crockett E, Evans L, Appleton B (Princess Of Wales Hospital, Bridgend); Griffiths E*, Dada O, Kulkarni R (Queen Elizabeth Hospital Birmingham); Albirnawi H*, Gravestock P, Vincenti C, Taribagil S, Dent B (Queen Elizabeth Hospital (Gateshead)); Tse C*, Clayton B, Burdekin E, Bannister L, Alam I (Royal Albert Edward Infirmary); Gray J*, Mactier M, Pollock A, Gough V (Royal Alexandra Hospital); Kanchustambam SR*, Ridgway M, Arujunan K (Royal Blackburn Teaching Hospital); Gopalswamy S*, Monteiro De Barros J, Lyons T, Griffith D (Royal Cornwall Hospital); Awan AK*, Latif J, Bandlamudi N, Bhatti I (Royal Derby Hospital); Raptis DA*, Machairas N, Pissanou T, Mestre-Costa J, Hidalgo Salinas C, Pollok JM (Royal Free Hospital); Al-Ardah M*, White A, Watson-Jones E, Rontree-Carey T, Boyce T (Royal Gwent Hospital); Hawkin P*, Elmaradny A, Ross K, Adu-Peprah E, Pinto K (Royal Lancaster Infirmary); Dunne D*, Mccready R, Nita G, Szatmary P, Tay VL, Rajput K (Royal Liverpool And Broadgreen Hospital); Rajendran I*, Chaudhury M, Zambas G (Royal Preston Hospital); Swaminathan C*, Atif QAA, Barrow T, Williams O, Malik A (Royal Sussex County Hospital); Conroy S*, Lindley S, Gilmore K, Boden E, Richards SK (Royal United Hospital); Hraishawi I*, Polak P, Mclaughlin D, Deeny D, Shuttleworth R, Harris A (Royal Victoria Hospital, Belfast); Peilober-Richardson A*, Morris GC, Sara X, Almourad H, Ang Y (Salford Royal Foundation Trust); Smyth R*, Ding D, Foster J, Bond A (Salisbury District Hospital); Kumar Y*, Ahmad A, Radoi D, Alkaili-Alyamani A, Balakrishnan S (Sandwell General Hospital); Satchidanand RY, Danwaththa Liyanage AS*, Blake I, Ransome M, Weerasinghe C (Southport And Formby District General Hospital); Kenington C*, Mayo K, Mohammed M (St Georges Hospital); Cockbain AJ*, Peckham-Cooper A, Mccauley G, Gordon C, Smith A (St James’s University Hospital); Hawkins W*, Chakravartty S, Baillie C, Kenny R (St Richards Hospital); Kumar A*, Koimtzis G, Bellamy E, Menon A (Stepping Hill Hospital, Stockport Nhs Foundation Trust); Kanakala A*, Nevins EJ, Madhavan A, Thulasiraman S, France K (The James Cook University Hospital); O’Connor A*, Idama D, Raslan C, Sridhar S, Parveen M (The Royal Oldham Hospital); Mubashar T*, Jarvis S, Cakmak I, Wright C, Andrews S (Torbay Hospital); Abdelsaid K* Abdul Aal Y, Jayasankar B, Morilla J, Shehata M, Subba N (Tunbridge Wells Hospital); Tewari N*, El-Sayed C, Somaie D, Beheiry N, Douka E (University Hospital Coventry); Arumugam S*, Wijetunga I, Leivers E, Ibrahim B, Khan K (University Hospital Of North Durham); Wheat J*, Christopher J, Barnett R (University Hospital Of Wales); Elberm H*, Booker J, Ashai S, Berry D (University Hospital Southampton); Luhmann A*, Sgro A, Rashid MM, Galea M, Jeyakumar J (Victoria Hospital Kirkcaldy); Marriott P*, Zafar S, Baker A, Yershov D, Galanopoulos G (Warwick Hospital); Gupta A*, Jordan R, Peinado Garcia C, Anyaugo N (Weston General Hospital); Bath MF*, Evans J, Omatseye J, Roberts L, Argyriou EO, Machesney M (Whipps Cross Hospital); Parmar C*, Clark S, Khalil H, Unsworth S (Whittington Health); Mlotshwa M*, Ayoub N, Aboelkhair A, Iosif E, Mohamed N (Worthing Hospital); Reynolds E*, Mackender E, Robinson D, Mufti W (Wythenshawe Hospital).

**United States of America (the) (US):** Fischkoff K*, Coleman N (Columbia University Irving Medical Centre); Anantha Sathyanarayana S*, Deutsch G, Giangola M, Lin D, Weiss M (Donald And Barbara Zucker School Of Medicine At Hofstra/Northwell); Chung C*, Nguyen A, Mueller J, Dabit M, Gordon J, McGuire E (HCA Swedish Medical Center); Rashid O*, Georgi E, Gallo M (Holy Cross Hospital); Kunstman JW*, Peters NV, O’Connor R, Bhattacharya B (Yale School of Medicine); Onkendi E*, Santos AP, Richmond R, Warren M, Zhang K (Texas Tech University Health Sciences Center); Broderick R*, Clary B, Horgan S, Doucet J, Liepert A (UC San Diego Health); Harmon L*, McCall C (University Of Colorado Hospital); Sham JG*, Williams E, Labadie KP, Clark NM, Dickerson LK (University Of Washington Medical Centre); Hammill CW*, Williams G, Kushner B, Cos H, Zarate Rodriguez J (Washington University In St. Louis); Bailey K* (West Virginia University Medical Corporation).

## Yemen (YE): Al-Raimi IMN*, Al-Zazay K, Ahmed Mohammed Al-Mahdi S, Mohammed Aldowbli S (Al-Khamseen Hospital); Al-Shehari M*, Shream S, Al-Ameri S, Aeed M, Al-Naggar H (Al-Thawra Modern General Hospital, Sana’a); Aldawbali M*, Alsayadi R, Alsayadi M (Royal Hospital).
